# Application of *Quercus pubescens* Acorn Flour and Xanthan Gum in Gluten-Free Cookies: RSM Optimization and Quality Evaluation

**DOI:** 10.3390/foods15050966

**Published:** 2026-03-09

**Authors:** Jasmina Lukinac, Dragana Medaković, Daliborka Koceva Komlenić, Ana Šušak, Marko Jukić

**Affiliations:** Faculty of Food Technology Osijek, Josip Juraj Strossmayer University of Osijek, Franje Kuhaca 18, 31000 Osijek, Croatia; dragana.m0799@gmail.com (D.M.); daliborka.koceva@ptfos.hr (D.K.K.); ana.susak@ptfos.hr (A.Š.); marko.jukic@ptfos.hr (M.J.)

**Keywords:** gluten-free cookies, acorn flour, *Quercus pubescens*, response surface methodology, xanthan gum, underutilized plant resources

## Abstract

Despite the growing demand for functional gluten-free (GF) foods, the application of *Quercus pubescens* acorn flour remains largely underexplored. This study addresses this gap by optimizing GF cookies using response surface methodology (RSM) and prepared with *Q. pubescens* acorn flour and xanthan gum to balance technological quality, sensory acceptability, and functional value. A three-level full factorial design (FFD) evaluated the effects of acorn flour proportion (0, 50 and 100%), and xanthan gum level (1, 2 and 3%) on physicochemical properties (moisture, water activity, color, texture, and dimensions), sensory attributes using a 9-point hedonic scale, proximate composition, and bioactive and antioxidant properties (total polyphenols, tannins, DPPH, ABTS, FRAP). Linear and quadratic polynomial models adequately described the experimental data (*R*^2^ = 0.86–0.99; non-significant lack of fit). Increasing acorn flour content significantly intensified cookie darkening, reduced snapping force and bending stiffness, reduced spread factor, and affected sensory perception, while xanthan gum improved structural integrity and dimensional stability. Multi-response optimization identified an optimal formulation containing 41.05% acorn flour and 1.46% xanthan gum, achieving balanced color development (darkness index ≈ 62), bending stiffness (~38 N/mm), and high overall sensory acceptability (~7.8). The optimized GF cookies exhibited a favorable nutritional profile and antioxidant properties, characterized by elevated total polyphenol content and antioxidant capacity, confirming the functional potential of acorn flour. The optimized cookies (containing 41.05% acorn flour) exhibited a six-fold increase in total phenolic content (from 1.63 to 10.08 mg GAE/g) and 8–10 times higher antioxidant capacity (DPPH, ABTS, and FRAP assays) compared to the control, confirming the substantial functional potential of *Q. pubescens* in gluten-free systems.

## 1. Introduction

There are over 500 species of oak worldwide, several of which have historically been consumed as part of the human diet [1]. The acorn, the fruit of oaks, has served as an important food resource for thousands of years in Europe, the Middle East, North Africa, Asia, and North America [2,3]. They were traditionally processed into flour and used for the preparation of bread, porridges, and soups [3,4], or roasted as a coffee substitute [1]. With the modernization of food systems and improved access to cultivated cereals, the use of acorns for human consumption declined significantly after World War II. Today, acorns are largely underutilized and are mainly used for animal feed, despite their nutritional and functional potential [1].

One of the main limitations of acorn utilization is their high tannin content, which imparts bitterness and astringency and may reduce nutrient bioavailability [5,6,7]. Historically, this challenge was addressed by soaking, roasting, or other traditional processing methods to reduce tannins [7,8]. Nevertheless, modern food processing methods allow controlled incorporation of acorn flour into food products, especially when sensory acceptance is carefully managed.

*Quercus pubescens* Willd., or pubescent oak, is widespread in central and southern Europe. Despite its abundance and ecological importance, its acorns are rarely used in modern human nutrition [9,10,11]. The species thus represents a largely untapped resource for developing value-added food products.

Recently, interest in acorns has resurged due to their nutritional value and potential as a sustainable functional food ingredient [1,12,13]. Acorn flour is rich in carbohydrates, dietary fiber, minerals, unsaturated fatty acids, and phenolic compounds [6,14,15,16,17]. They are rich in minerals (like Ca, Fe, Mg, K and P), provide vitamins A (provitamin A carotenoids) and E, and exhibit antioxidant, anti-inflammatory, and cardioprotective activities [18,19,20,21]. However, the composition strongly depends on the oak species, environmental conditions, and processing methods.

Importantly, acorns are naturally gluten-free. This makes acorn flour a promising ingredient for gluten-free product development, a sector that continues to grow due to medical and lifestyle-driven demand [16,17,22]. Gluten-free baked products often have inferior texture, reduced structure, and limited nutritional quality compared to their wheat-based counterparts. Cookies are especially suitable for gluten-free formulations [22,23]. Unlike bread or cakes, cookies depend less on gluten for structure and are therefore more tolerant of alternative flours. Rice flour and maize starch are commonly used gluten-free bases because of their neutral flavor and technological performance [24,25,26]. However, these ingredients offer limited nutritional value and functional benefits.

Compared to commonly used gluten-free enrichments such as legume flours (e.g., chickpea, lentil, soybean) and nut flours (e.g., almond, hazelnut), acorn flour differs not only in its macronutrient profile but also in its techno-functional behavior in bakery systems. Legume flours are primarily incorporated to enhance protein content and improve amino acid balance; however, they may impart characteristic beany flavors and increase dough density due to their high protein and fiber content [27,28]. Nut flours, while rich in lipids and contributing desirable sensory attributes, may increase product friability, caloric density, and affect structural stability [27,28].

In contrast, acorn flour is characterized by a higher proportion of complex carbohydrates, relevant amounts of unsaturated fatty acids, essential minerals, and a distinctive phenolic profile that contributes to antioxidant activity. However, its high fiber and phenolic content may interfere with starch gelatinization, water distribution, and matrix continuity in gluten-free systems, similar to other nutrient-dense non-cereal flours [27]. Therefore, incorporating such functional ingredients requires precise structural optimization.

Recent gluten-free formulation research increasingly emphasizes hydrocolloid optimization as a key factor in compensating for the absence of gluten and stabilizing complex matrices enriched with alternative flours. Hydrocolloids such as xanthan gum, hydroxypropyl methylcellulose (HPMC), psyllium, and guar gum play a crucial role in improving gas retention, dough viscoelasticity, crumb structure, and sensory quality [29,30,31]. Comparative studies have shown that the type and concentration of hydrocolloid significantly affect texture, loaf volume, and moisture retention in gluten-free bread and baked goods [32,33].

Moreover, optimization strategies based on response surface methodology (RSM) and mixture design have been successfully applied to gluten-free formulations enriched with legume and pseudo-cereal flours, enabling simultaneous evaluation of ingredient interactions and quality parameters [28,34]. Studies involving rice–soy flour systems and quinoa-based formulations further demonstrate that xanthan gum concentration must be carefully balanced to avoid excessive hardness or gumminess while maintaining structural integrity [30,35].

Despite these advances, systematic optimization of hydrocolloid levels in gluten-free cookies enriched with *Quercus pubescens* acorn flour remains insufficiently explored. Given the compositional particularities of acorn flour and its high phenolic and fiber content, targeted hydrocolloid adjustment is essential to achieve acceptable physicochemical and sensory quality. Hydrocolloids, such as xanthan gum, are often added to improve dough handling, structure, and texture in gluten-free systems. Xanthan gum increases water binding, viscosity, and matrix continuity, partially compensating for the lack of gluten [26,36]. Its effectiveness depends greatly on its concentration and interactions with other ingredients.

The incorporation of acorn flour remains underexplored, particularly for *Quercus pubescens*. Given its abundance, resilience, and ecological benefits, acorn flour represents a sustainable alternative to conventional crops while supporting rural economies and promoting forest biodiversity [1,9,37]. Furthermore, adding acorn flour into gluten-free formulations may simultaneously enhance nutritional quality and promote sustainability.

RSM is an effective statistical tool for optimizing complex food formulations. It enables simultaneous evaluation of multiple formulation variables and their interactions while minimizing experimental effort.

Therefore, the aim of this study was to optimize gluten-free cookie formulations containing *Quercus pubescens* acorn flour and xanthan gum using RSM. The effects of acorn flour proportion and xanthan gum level on physicochemical properties, sensory acceptability, nutritional composition, and antioxidant potential were systematically evaluated. The study aims to define an optimized formulation that enables meaningful incorporation of acorn flour without compromising product quality.

## 2. Materials and Methods

### 2.1. Materials

Rice flour (Nutrigold, Zagreb, Croatia) contained 12.2% moisture, 77.9% carbohydrates, 0.91% fat, 8.24% protein, and 0.75% ash. Maize flour (Dr. August Oetker KG, Bielefeld, Germany) contained 12.8% moisture, 72.5% carbohydrates, 3.3% fat, 9.75% protein, and 1.65% ash. Acorn flour (*Quercus pubescens*) contained 7.9% moisture, 78.29% carbohydrates, 5.4% fat, 6.5% protein, and 1.91% ash. Whey concentrate (SFD Nutrition, Opole, Poland) contained 73% protein; xanthan gum (Nutrimed, Zagreb, Croatia); margarine (Zvijezda plus Ltd., Zagreb, Croatia); sugar (sucrose) (Viro d.d., Virovitica, Croatia); salt (NaCl) (Solana Pag d.o.o., Pag, Croatia); leavening agents (sodium hydrogencarbonate (NaHCO_3_); and ammonium hydrogencarbonate (NH_4_HCO_3_)) (Pharmagal, Zagreb, Croatia).

### 2.2. Methods

#### 2.2.1. Preparation of Acorn Flour

**Figure 1:**
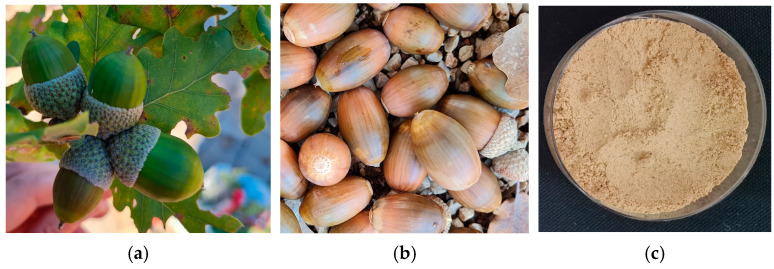
Morphological appearance of *Quercus pubescens* fruits and derived product used in the study: (**a**) immature acorns; (**b**) mature acorns selected for processing; (**c**) resulting acorn flour.

Acorns (*Quercus pubescens*) (Figure 1a,b) were collected during the 2024 harvest season at the Brzac location on the Island of Krk, Croatia (geolocation: 45.088901° N, 14.439238° E). Only mature and visually intact acorns were selected for further processing.

After collection, acorns were immersed in cold water to separate healthy fruits from damaged or infested ones. The selected acorns were dried in a laboratory oven (B40, Memmert GmbH + Co. KG, Schwabach, Germany) at 40 °C for 24 h. To facilitate shell removal, the dried acorns were slit longitudinally with a mechanical cutter (BL-CP-18, IRISLEE, Xingtai, China). After a second drying step at 40 °C for 24 h, the shells were manually removed. Residual testa was separated using a vibrating sieve (Fritsch GmbH–Mahlen und Messen, Model Analysette 3 PRO, Idar-Oberstein, Germany). No traditional debittering methods (such as leaching in water) or high-temperature roasting were performed. This processing strategy was chosen to preserve the natural antioxidant profile of *Q. pubescens* acorns. The removal of the testa (seed coat) using a vibrating sieve served as the primary physical method for reducing excessive astringency, as this fraction contains the highest concentration of condensed tannins. The two-stage drying at 40 °C allowed for controlled moisture reduction while maintaining the stability of the bioactive phenolic pool. The endosperm was milled using an ultracentrifugal laboratory mill (ZM 200, Retsch GmbH, Haan, Germany), and sieved to obtain particles smaller than 250 µm. The resulting acorn flour (Figure 1c) was stored under refrigeration until further analysis.

#### 2.2.2. Experimental Design and Preparation of Gluten-Free Acorn Cookies

RSM was used to evaluate the combined effects of formulation variables on the quality of gluten-free acorn cookies, as described by Jukić et al. (2024) [38]. A three-level FFD systematically assessed the influence of acorn flour (*A*) and xanthan gum (*B*) on multiple response variables. The design and data analysis were performed using Design-Expert^®^ software (version 12, Stat-Ease Inc., Minneapolis, MN, USA).

Two independent variables were considered: the proportion of acorn flour in the gluten-free flour blend (0%, 50%, and 100%) and the level of xanthan gum (1%, 2%, and 3%). The factor ranges were determined based on preliminary technological trials and the statistical requirements of the experimental design. For acorn flour (0–100%), preliminary tests confirmed that a bakeable matrix could be formed across the entire substitution range. For xanthan gum (1–3%), the range was selected based on our experience and previous research on gluten-free cookies [38]. Rice flour and maize flour were adjusted to maintain a constant total flour content. All other ingredients were kept constant and expressed as baker’s percentages (Table 1).

The experimental plan included twelve runs. Four of these runs (Runs 5, 10, 11, and 12) are replicates of the central point (50% acorn flour, 2% xanthan gum). Including multiple replicates at the center is standard in RSM designs and is necessary to estimate experimental (pure) error and to evaluate model adequacy through the lack-of-fit test. The order of experiments was randomized to minimize the influence of uncontrolled factors.

The evaluated responses included physical properties (CIEL*a*b* color parameters and darkness index), instrumental texture properties (snapping force, distance to break, and bending stiffness), dimensional characteristics (width, thickness, and spread factor), water content, water activity, and sensory attributes (appearance, color, texture, odor, taste, and overall acceptability).

The cookies were prepared according to the experimental design (Table 1). After weighing all ingredients as baker’s percentages (Table 1), margarine (40%), sucrose (42%), NaCl (1.25%), xanthan gum (1–3%), NaHCO_3_ (1%), and NH_4_HCO_3_ (1%) were mixed for 3 min at low speed using an electronic mixer (Gorenje MMC800W, Velenje, Slovenia). Water (22%) was then added, and mixing continued for 1 min at low speed, followed by 1 min at medium speed. The gluten-free flour mixture (100%), consisting of acorn flour, rice flour, and maize flour according to the experimental design, along with whey protein concentrate (10%), was then incorporated and mixed at low speed for 2 min.

The resulting dough was rounded, placed in a sealed PVC bag, and rested at 8 °C for 30 min. After resting, the dough was sheeted to a thickness of 5 mm and cut into circular pieces with a diameter of 45 mm. Baking was performed in a convection oven (Decktop XEKDT-01EU-S, Unox, Cadoneghe, Italy) at 180 °C for 14 min. The baked cookies were cooled at room temperature for 1 h before further analysis.

All baking trials were conducted in three independent batches, and all post-baking analyses were performed in triplicate using cookies from each batch.

### 2.3. Cookies Quality Evaluation

#### 2.3.1. Dimensions, Texture and Color Evaluation

Cookie dimensions and spread factor (*SF*) were determined in accordance with AACC Method 10-50D [39]. Six randomly chosen cookies were sampled from each of three independent batches (18 cookies per formulation), and average width (*W*, cm) and thickness (*H*, cm) were calculated. The *SF* was calculated as

(1) $$SF=\frac{W}{H}\cdot 10,$$

Texture properties of the acorn cookies, including snapping force (N), distance to break (mm), and bending stiffness (N/mm), were determined by a three-point bending test using a Shimadzu EZ-LX texture analyzer (Shimadzu Corp., Kyoto, Japan) equipped with a 1000 N load cell, following the procedure described by Jukić et al. (2024) [38] with minor modifications. Cookies were placed on two lower supports set 25 mm apart, with a knife blade positioned centrally above each sample. The blade moved downward at a constant speed of 1 mm/s until fracture occurred. Snapping force was recorded as the maximum force required to break the cookie. Distance to break was defined as the vertical displacement of the blade at the point of fracture, representing the distance at which the cookie breaks. Bending stiffness was calculated as the ratio of snapping force to distance to break. For texture evaluation, five randomly chosen cookies were sampled from each of three independent batches, totaling 15 cookies per formulation.

The surface color of acorn cookies was measured using an LS175 colorimeter (Hangzhou Lohand, Hangzhou, China) with a 20 mm measurement aperture. Measurements were performed under a 45/0 illumination geometry, with a D65 light source and a 10° standard observer. The results are expressed in the CIEL*a*b* color system. The achromatic component L* represents the brightness of the sample. The chromatic components are the green–red a* axis (ranging from −128 to +127) and the blue–yellow b* axis (ranging from −128 to +127) of the color space. The overall color change was expressed using the darkness index (*DI*) [40]:

(2) $$DI=\sqrt{{\left(100-L*\right)}^{2}+{\left(a*\right)}^{2}+{\left(b*\right)}^{2}},$$

The instrument was calibrated prior to each measurement using a standard white reference tile. For color evaluation, three cookies were sampled from each of three independent batches, totaling nine cookies per formulation. Color measurements were taken at five different locations on each cookie surface at 25 °C.

#### 2.3.2. Sensory Analysis of Acorn Cookies

Sensory analysis of acorn cookies was conducted by a panel of assessors with prior experience in sensory testing. The panel consisted of 11 women and 9 men, with an average age of 34 years. Assessors included staff and students of the Faculty of Food Technology Osijek who regularly participate in sensory evaluation as part of academic and research activities. The sensory assessment was conducted as a preliminary evaluation to support formulation optimization and comparative assessment of experimental samples, rather than as a population-based consumer acceptance study.

Inclusion criteria for panelists were the absence of medical conditions that could affect sensory evaluation (such as anosmia or color blindness) with no reported aversion to gluten-free bakery products. Sensory properties were rated on a 9-point hedonic scale, with scores ranging from 1 to 9 as follows: strongly dislike (1), dislike (2), moderately dislike (3), slightly dislike (4), neither like nor dislike (5), slightly like (6), moderately like (7), strongly like (8), and extremely like (9). The sensory properties evaluated were external appearance, texture, taste, and aroma. For recipe optimization, the overall sensory score was used as one of the response variables, determined by calculating the average of the scores for the sensory properties listed above. In addition to hedonic scoring, assessors were asked to provide qualitative comments on specific sensory characteristics, particularly bitterness, astringency, and nutty flavor notes as part of the taste scores. This allowed for a more detailed interpretation of changes in hedonic scores, especially regarding the characteristic flavors introduced by acorn flour.

Sensory analysis was conducted in a dedicated tasting room equipped with individual testing booths under controlled neutral white lighting (6500 K) to ensure consistent color perception [41]. Sensory evaluations were conducted during mid-morning hours under controlled environmental conditions to minimize potential variability related to circadian influences on sensory perception. All samples of gluten-free cookies were coded with random three-digit numbers and presented simultaneously. Sensory panel members were instructed to rinse their mouths with water between tastings. The panel members signed informed consent in accordance with European Union guidelines for ethics and food-related research [42]. To familiarize themselves with the study and the samples to be evaluated, the sensory panel members received a brief introduction to the research.

### 2.4. Extraction of Phenolic Compounds

Before chemical and antioxidant analyses, the baked cookies were ground into a fine powder using a knife mill (Grindomix GM200, Retsch GmbH, Haan, Germany) to ensure sample homogeneity. The equipment operated at 10,000 min^−1^ to achieve a final fineness of less than 200 µm, which is optimal for efficient extraction of bioactive compounds from the fatty cookie matrix. The ground cookies were stored in airtight containers at −18 °C until analysis. Ground cookies (10 mg) were weighed using an analytical balance (AB204-S, Mettler-Toledo GmbH, Greifensee, Switzerland). Then, 10 mL of solvent was added to each sample. A demineralized water was used for extraction [43,44,45]. After the solvent was added, the samples were mixed thoroughly with a vortex mixer (Vibromix 10, Domel Tehtnica d.o.o., Železniki, Slovenia). The samples were then placed in an ultrasonic water bath (Digital Pro+, Vevor, Shanghai, China) set at 30 °C, 40 kHz for 40 min to accelerate extraction. After this period, the samples were centrifuged (Multifuge 3L-R, Heraeus Instruments GmbH, Hanau, Germany) at 4000× *g* for 10 min. The supernatants containing polyphenols (total polyphenol extract—TPE) were immediately collected and used for further analysis. Prepared extract was used for the TPC (total polyphenol content) determination. A 5 mL volume of the polyphenol-rich supernatant was mixed with 50 mg of polyvinylpolypyrrolidone (PVPP). The mixture was left to stand, with occasional shaking every 5 min to facilitate tannin binding [46,47]. The samples were then centrifuged at 4000× *g* for 10 min. The supernatants, now depleted of tannins (TPE-PVPP), were carefully collected and immediately prepared for subsequent analyses.

### 2.5. Determination of Total Polyphenol Content, Non-Tannin Phenolic Compounds and Tannin Content

Polyphenol content was measured using the Folin–Ciocalteu method [48]. Briefly, 100 µL of TPE or TPE-PVPP was mixed with 100 µL of Folin–Ciocalteu reagent and incubated for 5 min in the dark. Then, 900 µL of sodium carbonate solution and 900 µL of distilled water were added. After 30 min of incubation in the dark, absorbance was measured at 765 nm using a spectrophotometer (UV-1280, Shimadzu Corporation, Kyoto, Japan). Results were expressed as gallic acid equivalents (GAEs) based on the calibration curve for gallic acid. Tannin content was calculated as difference between TPC and non-tannin phenolic compounds (NTPCs).

### 2.6. In Vitro Antioxidant Capacity

Extracts were prepared as described in Section 2.4.

#### 2.6.1. DPPH Radical Scavenging Activity

The radical scavenging activity of acorn cookies was evaluated using the DPPH method with minor modifications [49]. An aliquot of 100 µL of the aqueous extract of TPE was mixed with 3.9 mL of DPPH reagent. After 30 min of incubation in the dark, absorbance was measured at 517 nm. *DPPH* *inhibition* was calculated as

(3) $$\%\;DPPH\;inhibition\;=\frac{\left(A_{0}-A_{sample}\right)}{A_{0}}\times 100,$$

where *A*_0_ is the control absorbance and *A_sample_* is the absorbance of the test sample.

The antioxidant activity of the extracts was expressed as Trolox equivalents (TEs), based on the calibration curve.

#### 2.6.2. ABTS Radical Scavenging Activity

The ability of acorn cookie extracts to decolorize ABTS^+^ radical cations was evaluated according to Re et al. (1999) [50] with minor modifications. A 7 mM ABTS solution was mixed with 2.45 mM potassium persulfate, and the mixture was kept in the dark for 12–16 h. The solution was then diluted to achieve an absorbance of approximately 0.700 at 734 nm. For the assay, 100 µL of TPE aqueous extract was mixed with 3.9 mL of the prepared ABTS reagent. After 6 min of incubation in the dark, the absorbance was measured at 734 nm. The percentage of *ABTS radical inhibition* was calculated using the formula:

(4) $$\%\;ABTS\;radical\;inhibition\;\;=\;\frac{\left(A_{0}-A_{sample}\right)}{A_{0}}\times 100,$$

where *A*_0_ is the control absorbance and *A_sample_* is the absorbance of the test sample. The antioxidant activity was expressed as Trolox equivalents, based on the calibration curve.

#### 2.6.3. Ferric Reducing Antioxidant Power (FRAP) Assay

The FRAP of acorn cookie extracts was measured according to the method of Benzie and Strain (1996) [51], with minor modifications. The FRAP reagent was prepared by combining 300 mM acetate buffer (pH 3.6), 10 mM TPTZ in 40 mM HCl, and 20 mM FeCl_3_·6H_2_O in a ratio of 10:1:1 (*v*/*v*/*v*). Then, 100 µL of aqueous TPE was mixed with 3 mL of FRAP reagent. The mixture was incubated in a thermostated water bath at 37 °C for 10 min. After incubation, absorbance was measured at 594 nm using a spectrophotometer, with the reagent as a reference. The *FRAP value* was determined using the equation:

(5) $$FRAP\;value\;=\;A_{sample}-A_{0},$$

where *A*_0_ represents the absorbance of the control and *A_sample_* is the absorbance of the test sample. Antioxidant activities were expressed as Trolox equivalents (TEs), using the standard calibration curve.

### 2.7. Determination of Chemical Composition

Chemical composition was determined according to the following official methods: moisture content (AOAC 44-15.02) [52]; crude fat by Soxhlet extraction (AOAC 920.39) [53]; protein content by Kjeldahl nitrogen × 6.25 (AOAC 979.09) [54]; total dietary fiber by enzymatic–gravimetric analysis (AOAC 991.43) [55]. Total carbohydrates were calculated by difference from moisture, ash, protein, and fat contents. Total sugars were determined according to Commission Regulation (EC) No 152/2009 [56], and water activity (*a_w_*) was measured using the HygroPalm AW1 indicator (Rotronic AG, Bassersdorf, Switzerland) in ground samples of the acorn cookies [ISO 18787] [57]. Chemical composition and water activity (*a_w_*) were determined on homogeneously ground cookie samples prepared as described in Section 2.4. All measurements were performed in triplicate, using samples produced in three independent experimental batches.

### 2.8. Data Modeling and Optimization of Gluten-Free Acorn Cookies

Experimental data were analyzed using Design-Expert^®^ software (version 12.0.3.0, Stat-Ease Inc., Minneapolis, MN, USA). A three-level FFD was applied to evaluate the effects of acorn flour proportion (0, 50, and 100%) and xanthan gum level (1, 2, and 3%) on the quality of gluten-free acorn cookies. Four replicates at the central point were included to estimate experimental error and assess model adequacy.

RSM was used to model the relationships between the independent variables and all measured response variables. For each response, regression analysis was performed. The significance of model terms was evaluated by analysis of variance (ANOVA). For each response, the fitted regression equations and three-dimensional response surface plots were generated to describe and visualize the individual and interactive effects of the formulation variables.

Multi-response optimization was performed using the desirability function approach. Only selected responses were included in the optimization procedure: color (*DI*), texture (bending stiffness, N/mm), dimensional characteristics (spread factor), water activity (*a_w_*), and sensory quality (overall acceptability). Individual desirability functions (*D*) were defined for each selected response and scaled between 0 and 1, while the overall desirability was calculated as their geometric mean.

During optimization, overall acceptability was maximized, water activity was minimized, and target values were defined for the remaining responses (*DI* = 60.4, bending stiffness = 38 N/mm, and spread factor within the desired range). The optimal formulation was identified based on the maximum overall desirability value.

### 2.9. Statistical Analysis

All baking trials were conducted in three independent batches, and all post-baking analyses were performed in triplicate using cookies from each batch. Results are expressed as mean values ± standard deviation (*SD*). Statistical analysis was performed using Statistica^®^ software (version 14.0.0.15, TIBCO Software Inc., Palo Alto, CA, USA). The significance of the response surface models and their terms (RSM optimization) was evaluated by analysis of variance (ANOVA) using Design-Expert^®^ software. For the final comparison between the control cookies and the optimized acorn cookies (Section 3.4), an independent samples *t*-test was used to determine statistically significant differences. The level of statistical significance for all analyses was set at *p* < 0.05 (95% confidence level).

## 3. Results and Discussion

### 3.1. Model Adequacy and Statistical Validation

A RSM approach was used to evaluate the combined effects of acorn flour (*A*) and xanthan gum (*B*) on the color, textural, physical, and sensory properties of gluten-free acorn cookies. The adequacy of the fitted models was assessed using ANOVA, coefficients of determination, lack-of-fit tests, and diagnostic statistics, following established principles of experimental design and response surface modeling [58,59].

The significance of the quadratic terms (*A*^2^ and *B*^2^) for most physicochemical and sensory responses (Tables S1–S18) further justifies the chosen factor ranges. The presence of significant curvature within these boundaries confirms that the design space was appropriately sized to identify the optimal formulation.

ANOVA confirmed the statistical significance of the selected regression models (*p* < 0.05) for all responses, while the lack-of-fit tests were non-significant, indicating that the experimental data were adequately described by the proposed models within the studied experimental domain. A non-significant lack-of-fit is commonly regarded as a key indicator of model adequacy in RSM-based food formulation and optimization studies [28,59,60].

The coefficients of determination (*R*^2^), adjusted *R*^2^, and predicted *R*^2^ values showed good agreement (Table 2), indicating that a large proportion of the experimental variability was explained by the fitted models. The close correspondence between *R*^2^ and adjusted *R*^2^ further suggests that the regression equations were appropriately specified, without unnecessary model terms [58]. Specifically, the predicted *R*^2^ values (ranging from 0.4617 to 0.9949) confirm the models’ strong ability to predict new observations [34]. Furthermore, the low coefficient of variation (CV%) values, which remained below 4% for nearly all measured attributes, demonstrate the high precision and reproducibility of the experimental baking trials.

Adequate precision values exceeded the recommended minimum threshold of 4 for all responses, demonstrating an adequate signal-to-noise ratio and confirming that the models can be reliably used to explore and navigate the experimental design space [61].

The selection of linear or quadratic model forms was based on model hierarchy, statistical significance of regression terms, lack-of-fit results, and physical interpretability of the response behavior. Linear models were retained when no significant curvature was detected, indicating additive and proportional effects of the formulation variables. Quadratic models were selected when significant second-order terms revealed the curvature of the response surface and the presence of optimal regions within the experimental domain, which are characteristic of complex food systems optimized using RSM [59,60].

The high *R*^2^, adjusted *R*^2^, and predicted *R*^2^ values reported in Table 2, together with the non-significant lack-of-fit results (Tables S1–S18), confirm that the selected models adequately describe the relationships between formulation variables and the measured quality attributes of gluten-free acorn cookies and can be reliably used for subsequent multi-response optimization.

### 3.2. Effect of Acorn Flour and Xanthan Gum on Response Variables

#### 3.2.1. Effect of Acorn Flour and Xanthan Gum on Color Parameters and Darkness Index

**Figure 2:**
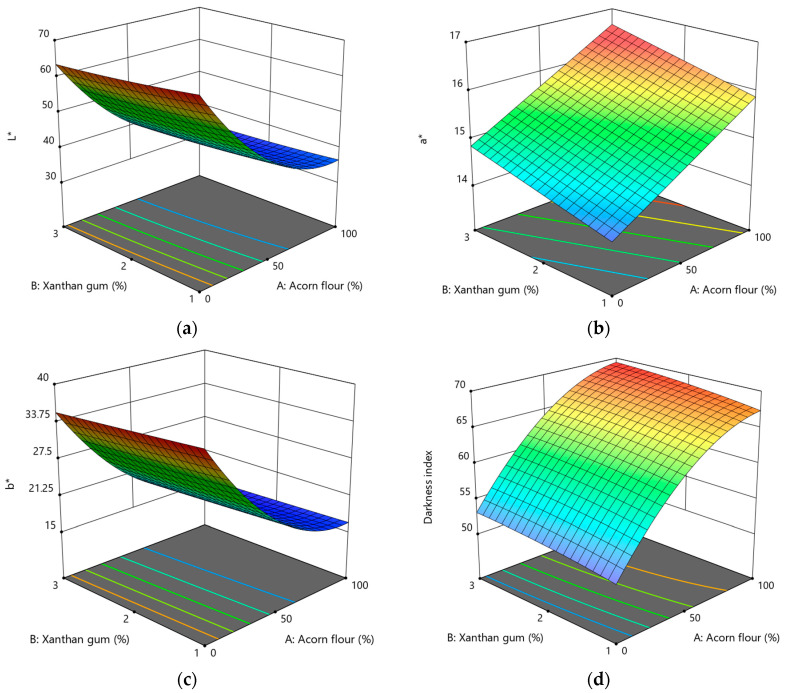
Response surface plots showing the effect of acorn flour (A) and xanthan gum (B) on color parameters and whiteness index of gluten-free acorn cookies: (**a**) L* (lightness); (**b**) a* (red–green); (**c**) b* (yellow–blue); (**d**) darkness index (*DI*).

The lightness (L*) values ranged from approximately 34.0 to 67.2, indicating pronounced differences in cookie surface color. The color of gluten-free acorn cookies was primarily determined by the proportion of acorn flour, with a secondary effect from xanthan gum. Increasing the acorn flour content caused a marked decrease in L*, reflecting progressive cookie darkening (Figure 2a). This response is well described by a quadratic model (Equation (6)), which was highly significant according to ANOVA (*p* < 0.0001, Table S1). Both the linear and quadratic terms for acorn flour were significant (*p* < 0.0001), while xanthan gum had a smaller but statistically significant linear effect (*p* = 0.0022). The interaction between factors was not significant (*p* = 0.2530). These results indicate an accelerated darkening effect at higher acorn flour levels.

(6) $$L*=41.31-14.83\cdot A-1.40\cdot B+0.43\cdot AB+8.71\cdot A^{2}+0.41\cdot B^{2},$$

The red–green coordinate (a*) ranged from approximately 14.0 to 16.4 and increased linearly with both acorn flour and xanthan gum content (Figure 2b). The linear regression model (Equation (7)) was statistically significant (*p* < 0.0001, Table S2), with both factors contributing positively (*A*: *p* < 0.0001; *B*: *p* = 0.0026). The absence of a significant interaction suggests an additive and predictable shift toward redder hues as formulation levels increase.

(7) a*=15.37+0.9·A+0.38·B,

The yellow–blue coordinate (b*) ranged from approximately 16.0 to 36.9 and decreased significantly as acorn flour content increased (Figure 2c). The quadratic model (Equation (8)) showed significant linear and quadratic effects of acorn flour (*p* < 0.0001), while xanthan gum and interaction effects were not significant (*p* > 0.05, Table S3). The reduction in b* values indicates a loss of yellow tones and a shift toward darker brown coloration, suggesting that color development in *Q. pubescens* acorn cookies is driven by browning reactions rather than increased yellowness.

(8) $$b*=20.65-9.98\cdot A-0.62\cdot B+0.23\cdot AB+5.54\cdot A^{2}+0.04\cdot B^{2},$$

The darkness index (*DI*) ranged from 51.3 to 69.5 and increased significantly with acorn flour content (Figure 2d), confirming the overall darkening trend. *DI* was well described by a quadratic model (Equation (9)). Acorn flour showed strong linear and quadratic effects (*p* < 0.0001), while xanthan gum had a secondary but statistically significant linear effect (*p* = 0.0055). The interaction effect was not significant (*p* = 0.8571), indicating that acorn flour is the dominant factor controlling cookie darkening.

(9) $$DI=64.1+8.09\cdot A+0.95\cdot B+0.05\cdot AB-3.5\cdot A^{2}-0.32\cdot B^{2},$$

Overall, color parameters were primarily affected by the proportion of acorn flour. Higher substitution levels produced darker cookies, as indicated by lower L* and b* values and higher *DI* and a* values. This pattern aligns with the natural pigmentation of acorn flour and the intensification of Maillard and phenolic oxidation reactions during baking [1,22,62]. Acorn flour is rich in phenolic compounds and reducing sugars, which actively participate in non-enzymatic browning reactions and contribute to red–brown color development. While some studies report simultaneous increases in a* and b* values in acorn-enriched products, the present results suggest that for *Q. pubescens* flour, browning reactions predominate over yellow pigment retention, resulting in a net reduction in b* values.

Xanthan gum affected color parameters to a lesser extent but showed statistically significant effects on L*, a*, and *DI*. These effects are likely indirect and related to changes in moisture distribution, dough viscosity, and heat transfer during baking, which can alter surface dehydration and browning kinetics. Similar secondary effects of hydrocolloids on color development in gluten-free cookies have been reported previously [63]. Despite the observed darkening, the resulting color remained within an acceptable range for cookies and was positively perceived during sensory evaluation.

#### 3.2.2. Effect of Acorn Flour and Xanthan Gum on Texture Properties

**Figure 3:**
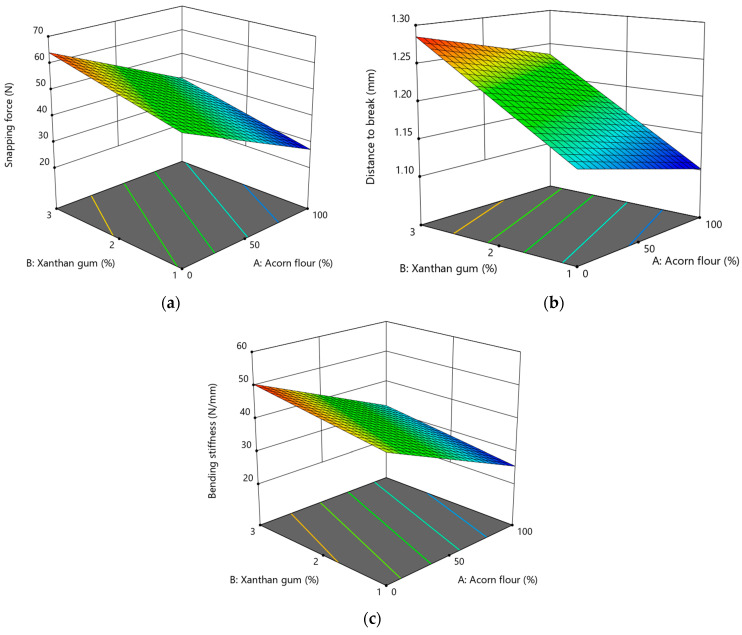
Response surface plots showing the effect of acorn flour (A) and xanthan gum (B) on texture properties of gluten-free acorn cookies: (**a**) snapping force; (**b**) distance to break; (**c**) bending stiffness.

Snapping force values ranged from 28.40 to 66.10 N and were significantly affected by both formulation variables (Figure 3a). Increasing the proportion of acorn flour caused a pronounced reduction in the force required to fracture the cookies, indicating a more fragile and brittle structure. This effect can be attributed to dilution of the continuous starch–protein matrix and the disruptive presence of dietary fiber particles, which interfere with stress transmission during bending and promote crack initiation. A similar decrease in fracture force with increasing acorn flour content has been reported for acorn-enriched biscuits by Pasqualone et al. (2019) [22].

In contrast, increasing the xanthan gum concentration significantly increased snapping force, reflecting enhanced internal cohesion and structural reinforcement. Xanthan gum likely forms a continuous hydrocolloid network that improves particle binding, increases dough viscosity, and enhances resistance to fracture. Similar strengthening effects of xanthan gum on the mechanical resistance and fracturability of gluten-free cookies have been reported by Gül et al. (2018) [63].

Snapping force was adequately described by a linear regression model (Equation (10)), showing a strong negative effect of acorn flour and a positive effect of xanthan gum (*p* < 0.0001, Table S5), with no significant interaction between the variables, indicating additive and independent contributions of both factors.

(10) Snapping force (N)=45.63−12.07·A+6.35·B,

The distance to break ranged from 1.11 to 1.29 mm and was primarily determined by xanthan gum content (Figure 3b). Higher xanthan gum levels increased deformation at fracture, indicating greater flexibility and ductility of the cookie matrix. Acorn flour had a weaker but statistically significant negative effect, consistent with the stiffening action of insoluble fibrous particles that limit deformation before fracture. The linear model (Equation (11)) confirmed a narrow response range and a dominant effect of xanthan gum (*p* < 0.0001, Table S6), indicating proportional and predictable changes within the studied formulation space.

(11) $$Distance\;to\;break\;\left(\mathrm{mm}\right)=1.19-0.03\cdot A+0.07\cdot B,$$

Bending stiffness values ranged from approximately 26 to 51 N/mm (Figure 3c). Increasing acorn flour content significantly reduced bending stiffness, indicating the formation of a weaker, less cohesive structure with limited load-bearing capacity. This result aligns with the reduced network-forming ability of acorn flour components and the mechanical discontinuities introduced by dietary fiber. In contrast, xanthan gum significantly increased bending stiffness, confirming its reinforcing role in gluten-free cookie systems by increasing dough viscosity and internal cohesion, as also reported by Gül et al. (2018) [63].

A linear model (Equation (12)) adequately described bending stiffness, with both factors showing significant effects (*p* < 0.0001, Table S7) and no evidence of interaction or curvature.

(12) $$Bending\;stiffness\;\left(N/\mathrm{mm}\right)=38.06-9.27\cdot A+3.2\cdot B,$$

Although higher acorn flour levels reduced the snapping force and bending stiffness, they were associated with greater resistance to initial deformation, resulting in a texture described as hard but brittle. In such structures, fibrous particles increase resistance during the early stages of loading but promote sudden fracture once critical stress is exceeded. Similar mechanical behavior has been reported for fiber-enriched cookie systems, where disruption of starch continuity increases brittleness and reduces fracture tolerance [64].

Xanthan gum improved textural uniformity and partially mitigated excessive brittleness at intermediate concentrations. This effect is linked to its strong water-binding capacity and its ability to enhance matrix continuity, thereby mimicking some structural functions of gluten and improving bite quality in gluten-free systems [65]. However, response surface analysis indicated that excessive xanthan gum addition adversely affected texture, likely due to over-gelation, excessive dough viscosity, and reduced cookie spread during baking, as previously observed in gluten-free cookies by Mancebo et al. (2015) [24].

#### 3.2.3. Effect of Acorn Flour and Xanthan Gum on Dimensions of Cookies

Physical dimensions are key indicators of dough behavior during baking and reflect formulation-induced changes in rheology, gas retention, and water distribution. In gluten-free cookie systems, where structural development is governed primarily by viscosity rather than elastic gluten networks, cookie width, thickness, and spread factor are particularly sensitive to ingredient composition (Pasqualone et al., 2019) [22].

**Figure 4:**
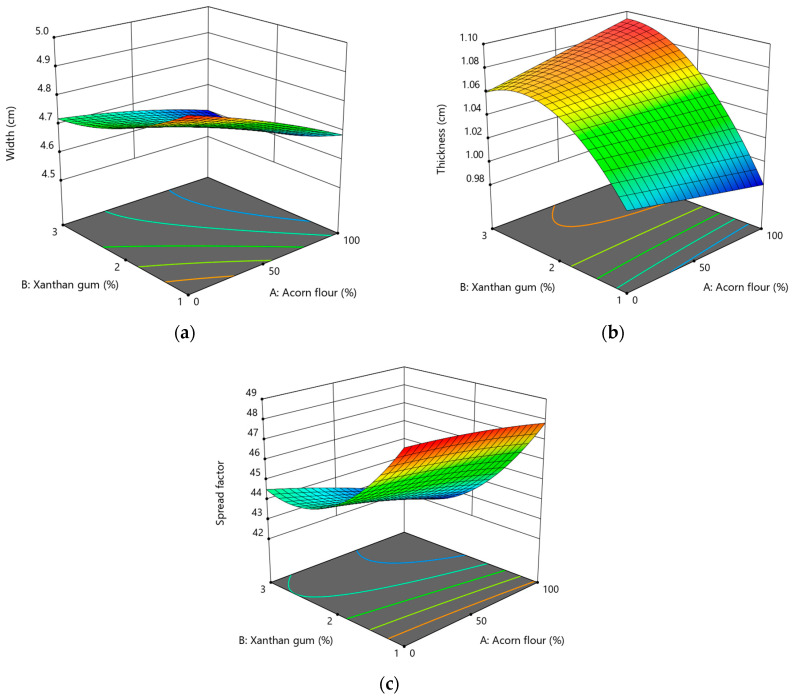
Response surface plots showing the effect of acorn flour (A) and xanthan gum (B) on physical properties of gluten-free acorn cookies: (**a**) width; (**b**) thickness; (**c**) spread factor.

Cookie width ranged from 4.60 to 4.90 cm. Increasing both acorn flour and xanthan gum levels significantly reduced cookie width (Figure 4a), indicating restricted dough spreading during baking. This behavior reflects limited dough flow, primarily attributed to the high dietary fiber content of acorn flour. Dietary fibers have high water absorption capacity, increasing dough viscosity and reducing the amount of free water available for sugar dissolution, a key factor promoting cookie spread [24,66]. Consequently, lateral expansion during baking is reduced.

Xanthan gum also had a significant narrowing effect on cookie width. Due to its strong water-binding ability and high molecular weight, xanthan gum markedly increases dough consistency even at low concentrations. Similar reductions in cookie diameter with increasing xanthan gum levels have been reported in gluten-free cookie formulations, where hydrocolloids act as dominant rheological modifiers [63]. Increased viscosity limits horizontal dough flow and promotes shape retention.

Cookie width was adequately described by a quadratic model (Equation (13)), with significant linear effects of both acorn flour (*p* < 0.0001) and xanthan gum (*p* = 0.0001), and a significant quadratic effect of xanthan gum (*p* = 0.0167, Table S8). A weak but significant interaction effect (*p* = 0.0364) indicates that the influence of xanthan gum on width slightly depends on the acorn flour level, although this interaction is not technologically dominant.

(13) $$Width\;\left(\mathrm{cm}\right)=4.70-0.08\cdot A-0.07\cdot B+0.03\cdot AB-0.01\cdot A^{2}+0.03\cdot B^{2},$$

Cookie thickness ranged from approximately 0.98 to 1.09 cm and was mainly affected by xanthan gum content (Figure 4b). Increasing xanthan gum levels resulted in thicker cookies, reflecting improved gas retention and reduced dough collapse during baking. Hydrocolloids are known to stabilize entrapped air and carbon dioxide bubbles, promoting vertical expansion rather than lateral flow [63].

The significant quadratic effect of xanthan gum suggests an optimal concentration range. At higher levels, excessive dough rigidity may limit further expansion, a phenomenon previously observed in hydrocolloid-enriched gluten-free dough systems [67]. In contrast, acorn flour did not significantly affect cookie thickness within the studied range (*p* = 0.8291, Table S9). This finding is consistent with previous research indicating that acorn flour primarily influences cookie diameter rather than thickness [22]. The limited impact on thickness suggests that acorn flour modifies dough flow mainly through compositional and hydration-driven effects rather than through mechanisms related to gas retention or matrix elasticity.

Cookie thickness followed a quadratic model (Equation (14)), with xanthan gum showing significant linear (*p* = 0.0013) and quadratic effects (*p* = 0.0353), while interaction and acorn flour effects were not significant (Table S9).

(14) $$Thickness\;\left(\mathrm{cm}\right)=1.07+0.002\cdot A+0.04\cdot B+0.02\cdot AB-0.03\cdot B^{2},$$

Spread factor: The spread factor ranged from 42.20 to 48.51 and decreased with increasing levels of both acorn flour and xanthan gum (Figure 4c), confirming restricted dough flow during baking. Xanthan gum had the strongest influence on spread behavior, consistent with its pronounced effect on dough viscosity. Doughs with higher viscosity exhibit reduced spread during baking, as widely reported for both wheat-based and gluten-free cookie systems [24,68].

The reduction in spread factor associated with acorn flour addition is attributed to its high water absorption capacity and complex composition, including dietary fiber, damaged starch, and lipids. While gluten dilution may increase spread in some formulations, interactions among flour components in gluten-free systems often lead to contrasting effects [22]. Similar reductions in cookie spread have been observed in formulations enriched with legume or nut flours characterized by high hydration properties [69].

The spread factor was adequately described by a quadratic model (Equation (15)). Xanthan gum showed a dominant effect, with significant linear (*p* = 0.0002) and quadratic (*p* = 0.0112) terms, while acorn flour exhibited a weaker but significant linear effect (*p* = 0.0409). The substantially higher sum of squares associated with xanthan gum confirms that spread behavior in gluten-free acorn cookies is primarily governed by hydrocolloid-induced viscosity rather than by flour substitution level (Table S10).

(15) $$Spread\;factor=44.2-0.83\cdot A-2.48\cdot B-0.39\cdot AB-0.13\cdot A^{2}+1.73\cdot B^{2},$$

Overall, cookie geometry in this gluten-free system was governed primarily by viscosity-controlled flow mechanisms rather than gluten-related elastic effects. Xanthan gum played a dominant role in controlling thickness and spread, while acorn flour mainly influenced cookie width through its fiber-rich and highly hydrophilic composition. Moderate levels of both ingredients produced cookies with balanced dimensions and acceptable shape, supporting their technological suitability in gluten-free cookie formulations.

Because cookie dough systems exhibit predominantly plastic behavior, physical indicators such as spread factor and dimensional changes during baking are commonly used as technological proxies for dough flow properties, rather than farinograph-type viscoelastic measurements.

#### 3.2.4. Effect of Acorn Flour and Xanthan Gum on Water Content and Water Activity

Water content and water activity are critical parameters that govern the texture, shelf stability, and overall quality of baked products. In gluten-free systems, these properties are especially sensitive to ingredient hydration capacity and water-binding mechanisms, as structure formation relies primarily on viscosity rather than gluten network development.

**Figure 5:**
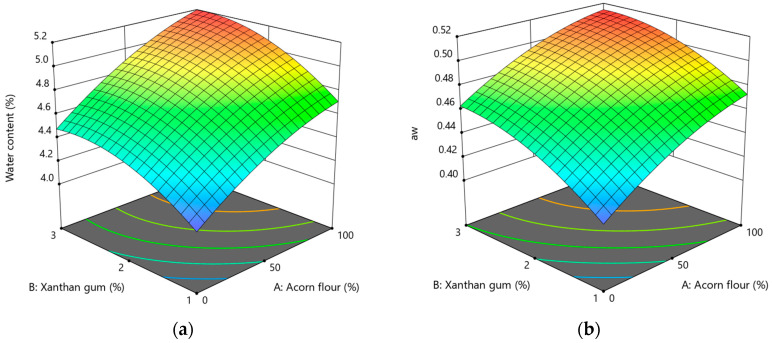
Response surface plots showing the effect of acorn flour (A) and xanthan gum (B) on water-related properties of gluten-free acorn cookies: (**a**) water content; (**b**) water activity (*a_w_*).

The water content of gluten-free acorn cookies ranged from 4.10% to 5.20% and increased significantly with higher levels of both acorn flour and xanthan gum (Figure 5a). This reflects the high water-binding capacity of both ingredients. Acorn flour is rich in dietary fiber and complex carbohydrates, which retain water through physical entrapment and hydrogen bonding [70,71]. Xanthan gum further enhances moisture retention due to its highly hydrophilic polysaccharide structure and high molecular weight, which promote water immobilization within the dough matrix.

Water content was described by a quadratic model (Equation (16)); however, ANOVA showed that the response was predominantly governed by significant linear effects of acorn flour (*p* = 0.0001) and xanthan gum (*p* = 0.0013), while quadratic and interaction terms were not statistically significant (Table S11). This indicates a mainly proportional increase in total moisture retention within the studied formulation range, with only a slight tendency toward saturation at higher substitution levels.

(16) $$Water\;content\;\left(\%\right)=4.85+0.33\cdot A+0.22\cdot B+0.03\cdot AB-0.09\cdot A^{2}-0.14\cdot B^{2},$$

***a_w_*** values ranged from 0.41 to 0.52 and were significantly influenced by both factors, showing a general increase with higher inclusion levels of acorn flour and xanthan gum (Figure 5b), indicating changes in water availability rather than total water content. Fiber-rich ingredients may increase *a_w_* despite higher moisture levels, as part of the absorbed water remains mobile within the matrix [72]. In contrast to water content, *a_w_* exhibited significant quadratic effects for both factors (*p* < 0.05, Table S12), suggesting a non-linear response (Equation (17)).

At lower xanthan gum levels, ***a_w_*** increased due to reduced water retention and redistribution within the matrix. However, at the highest concentration (3%), the significant quadratic effect (Table S12) indicates that the hydrocolloid network becomes more effective in immobilizing water molecules. This limits the further increase in *a_w_*, consistent with Gül et al. (2018) [63], who noted that elevated hydrocolloid levels stabilize water within the matrix, thereby controlling its availability. Despite the observed increases, all ***a_w_*** values remained well below thresholds associated with microbial growth, confirming that the hydration changes did not compromise microbiological stability [73].

(17) $$a_{w}=0.49+0.03\cdot A+0.02\cdot B-0.002\cdot AB-0.009\cdot A^{2}-0.01\cdot B^{2},$$

The combined effects of acorn flour and xanthan gum highlight the importance of balanced hydration in gluten-free cookie formulations. Acorn flour increases total water retention through its fibrous structure, while xanthan gum modulates water distribution and availability. Similar interactions between fiber-rich flours and hydrocolloids have been reported in gluten-free baked goods, where controlled moisture retention contributes to improved texture and shelf-life potential [74]. Overall, moderate incorporation levels of both ingredients allowed enhanced moisture retention while maintaining water activity within a technologically safe range. This balance supports both product stability and desirable textural properties in gluten-free acorn cookies.

#### 3.2.5. Effect of Acorn Flour and Xanthan Gum on Sensory Evaluation

**Figure 6:**
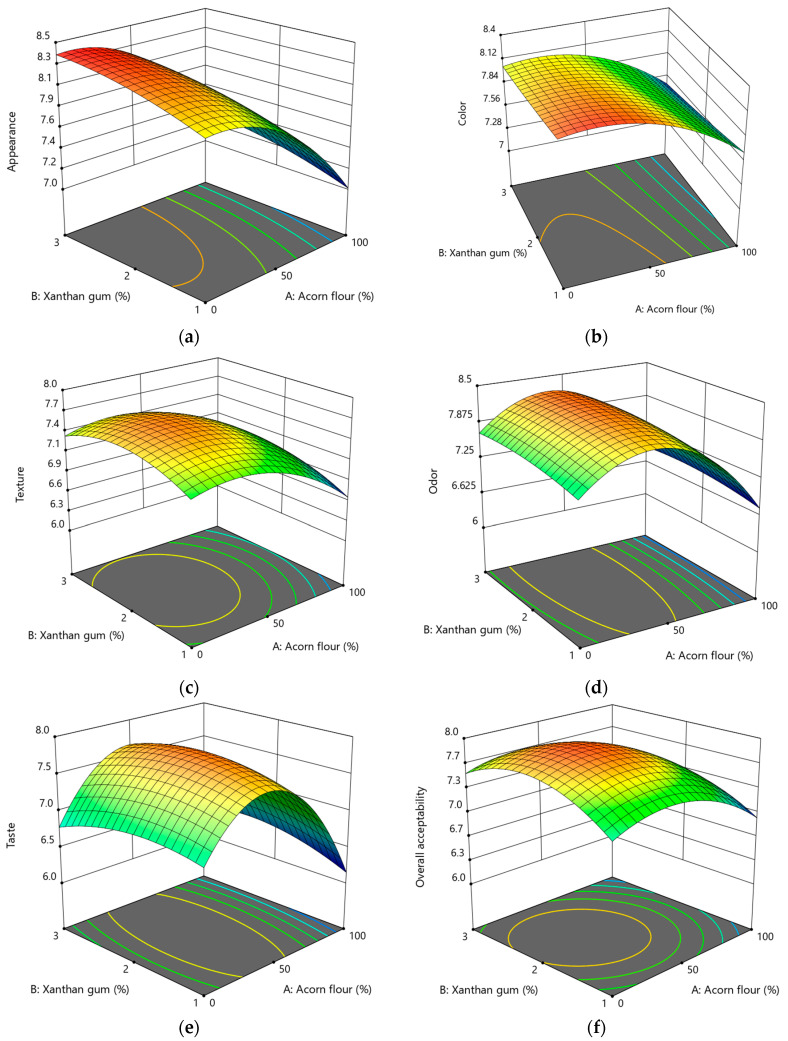
Response surface plots showing the effect of acorn flour (A) and xanthan gum (B) on sensory attributes of gluten-free acorn cookies: (**a**) appearance, (**b**) color, (**c**) texture, (**d**) odor, (**e**) taste, (**f**) overall acceptability.

Figure 6 shows the response surface plots describing the effects of acorn flour (A) and xanthan gum (B) on the sensory attributes of gluten-free acorn cookies, including appearance, color, texture, odor, taste, and overall acceptability.

Appearance scores ranged from 7.0 to 8.4 and decreased significantly with increasing acorn flour content, reflecting darker color development and reduced surface uniformity. Acorn flour had significant linear and quadratic effects (*p* < 0.0001 and *p* = 0.0010, respectively; Table S13), indicating that moderate substitution levels resulted in the highest appearance acceptance. Xanthan gum showed a mild but significant positive linear effect (*p* = 0.0032), likely due to improved dough cohesion and surface homogeneity, while its quadratic term and interaction with acorn flour were not significant. Appearance was adequately described by a quadratic model (Equation (18)).

(18) $$Appearance=8.07-0.52\cdot A+0.17\cdot B-0.3\cdot A^{2}-0.06\cdot B^{2},$$

Color acceptability scores ranged from approximately 7.4 to 8.3 and were mainly influenced by acorn flour content. Increasing substitution levels significantly reduced sensory color scores (*p* = 0.0010), consistent with instrumental color measurements (Figure 6b). Similar sensory responses have been reported for acorn-enriched biscuits, breads, and pasta, where moderate darkening was associated with a wholegrain or artisanal appearance [22,62,75]. Excessive darkening, however, reduced visual appeal, confirming the need for formulation optimization [24]. The significant quadratic term of acorn flour (*p* = 0.0158; Table S14) indicates an optimal intermediate level, while xanthan gum had no significant linear or quadratic effect on color perception (Equation (19)).

(19) $$Color=8.08-0.28\cdot A-0.1\cdot B-0.24\cdot A^{2}+0.013\cdot B^{2},$$

Texture scores ranged from 6.6 to 7.8 and were strongly affected by acorn flour content. Increasing substitution levels significantly reduced texture acceptability (*p* = 0.0005), probably due to the more friable and grainier structure which is consistent with instrumental texture measurements and previous reports on acorn-enriched cookies and gluten-free bakery products [22,24]. These effects are mainly attributed to the dietary fiber and lipid fractions of acorn flour, which disrupt the starch-based matrix and weaken structural cohesion. The significant quadratic effect of xanthan gum (*p* = 0.0111; Table S15, Equation (20)) suggests that intermediate concentrations partially compensated for this effect by improving water retention and matrix continuity, resulting in improved mouthfeel. Similar behavior has been observed in gluten-free cookies and acorn-based cakes [71,75].

(20) $$Texture=7.6-0.3\cdot A+0.07\cdot B+0.03\cdot AB-0.34\cdot A^{2}-0.24\cdot B^{2},$$

Odor scores ranged from approximately 6.8 to 8.4 and decreased with increasing acorn flour content. Both significant linear (*p* = 0.0006) and strong quadratic effects (*p* < 0.0001; Table S16, Equation (21)) were observed, indicating an optimal intermediate substitution level. Higher acorn flour concentrations intensified earthy and nut-like aromas characteristic of acorn flour, which were not always positively perceived. Comparable odor-related trends have been reported in acorn-enriched bakery products [22,75]. Xanthan gum did not significantly influence odor perception, consistent with its neutral sensory profile.

(21) $$Odor=8.2-0.38\cdot A+0.08\cdot B-0.89\cdot A^{2}-0.09\cdot B^{2},$$

Taste acceptability ranged from approximately 6.2 to 8.0 and was primarily governed by acorn flour content. Significant linear (*p* = 0.0008) and quadratic effects (*p* < 0.0001; Table S17, Equation (22)) indicated that moderate substitution levels were most acceptable. At higher concentrations, panelists noted that bitterness and astringency became more noticeable, effects commonly associated with phenolic compounds and tannins present in acorn flour [22,75]. Reduced sweetness perception was likely due to sugar dilution and flavor masking by phenolics, while caramel and roasted notes perceived at moderate levels may reflect enhanced Maillard reactions. Xanthan gum showed a significant quadratic effect (*p* = 0.0440), suggesting an indirect contribution to taste perception through improved oral lubrication and texture rather than direct flavor modification.

(22) $$Taste=7.79-0.28\cdot A+0.03\cdot B+0.13\cdot AB-1.02\cdot A^{2}-0.18\cdot B^{2},$$

Overall acceptability scores ranged from approximately 6.9 to 8.0 and followed a clear quadratic trend with respect to both formulation variables. Acorn flour showed significant linear and quadratic effects (*p* = 0.0005; Table S18, Equation (23)), confirming an optimal intermediate substitution level. Xanthan gum did not exhibit a significant linear effect; however, its quadratic term was highly significant (*p* = 0.0005), indicating that moderate concentrations enhanced the overall acceptability by improving the texture and structural integrity. Similar trends have been reported for acorn-enriched biscuits, breads, and cakes, where substitution levels up to approximately 30% maintained or improved sensory acceptance, while higher levels reduced acceptability [22,71,75,76].

(23) $$Overall\;acceptability=7.88-0.23\cdot A+0.07\cdot B-0.05\cdot AB-0.35\cdot A^{2}-0.35\cdot B^{2},$$

For all sensory attributes, acorn flour was the dominant factor influencing sensory quality, with significant linear and/or quadratic effects observed for all parameters. The highest sensory scores were consistently obtained at intermediate levels of acorn flour and xanthan gum, confirming that successful incorporation of acorn flour into gluten-free cookies requires careful control of the substitution level and appropriate use of structuring agents. When properly optimized, acorn flour can enhance the nutritional and functional profile of gluten-free cookies without compromising sensory quality.

### 3.3. Optimization of Gluten-Free Acorn Cookie Formulation

The quality of gluten-free acorn cookies is determined by the combined effects of formulation variables rather than by a single ingredient. Both acorn flour content and xanthan gum concentration significantly influenced the physicochemical properties (moisture, water activity, color, texture, and dimensions) and sensory attributes, although the magnitude and direction of these effects varied depending on the specific response. This is consistent with previous studies showing that fiber-rich flours and hydrocolloids interact synergistically to influence gluten-free cookie quality [22,63].

To ensure robust multi-response optimization, only representative responses were included based on three criteria: (i) high sensitivity to formulation variables, (ii) adequate model fit and statistical significance, and (iii) direct technological or sensory relevance to cookie quality.

Selected responses for optimization were darkness index (*DI*), bending stiffness (N/mm), spread factor, water activity (*a_w_*), and overall acceptability, with overall acceptability treated as an integrated sensory response encompassing appearance, texture, odor, and taste. Antioxidant responses (TPC, DPPH, ABTS, and FRAP) were not included in the multi-response desirability function because their enhancement was inherently determined by the substitution level of acorn flour. Since the optimization goal was to maximize the proportion of acorn flour, the functional potential was simultaneously optimized by proxy. This allowed the desirability function to focus specifically on balancing the technological and sensory attributes, which are the primary limiting factors when incorporating high-fiber and high-tannin resources into a gluten-free matrix. The subsequent 6- to 10-fold increase in bioactive potential observed in the optimized formulation validates this optimization strategy.

Acorn flour content was set to be maximized to enhance the nutritional and functional value, while xanthan gum was constrained to 1–3% to maintain dough integrity and control cookie spread. Target values for *DI* and bending stiffness were defined to achieve a balanced appearance and adequate structural strength, whereas water activity was minimized to improve shelf stability. Overall acceptability and acorn flour content were assigned the highest relative importance, reflecting the primary objective of maximizing nutritional enhancement without compromising consumer acceptance or technological performance.

**Figure 7:**
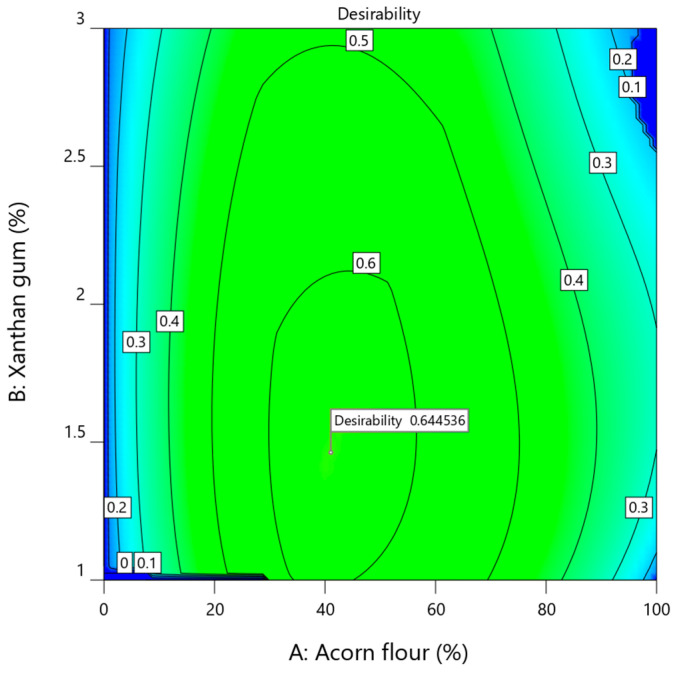
Overall desirability surface plot showing the combined effect of acorn flour (*A*) and xanthan gum (*B*) on multi-response optimization of gluten-free acorn cookies.

Multi-response optimization was conducted using the desirability function approach in Design-Expert software. Among the generated solutions, the formulation with the highest overall desirability (*D* = 0.645) was selected as the optimal compromise between nutritional, technological, and sensory criteria, corresponding to 41.05% acorn flour and 1.46% xanthan gum. The desirability surface (Figure 7) indicates that the highest desirability values occur at intermediate acorn flour levels combined with relatively low xanthan gum concentrations, representing a balance between color development, textural integrity, dimensional stability, controlled water activity, and sensory acceptance.

All observed responses were statistically consistent with the predicted values, as they fell within the 95% prediction intervals (PIs). No statistically significant differences were detected (*p* > 0.05, Student’s *t*-test), confirming the robustness and predictive power of the developed response surface models. The minor deviations observed for certain dimensional parameters, particularly spread factor, likely reflect the inherent variability in gluten-free dough handling and baking conditions, but did not compromise the overall product quality or acceptability.

These results demonstrate that acorn flour can be incorporated at moderate levels to improve the nutritional and functional potential of gluten-free cookies without adversely affecting the technological or sensory attributes. Similar optimization approaches have been successfully applied to other fiber-rich and functional flours in gluten-free bakery products [71,77]. The combination of acorn flour and xanthan gum allows for structural reinforcement, controlled spread, and enhanced mouthfeel, aligning with prior observations on hydrocolloid–flour interactions in gluten-free systems [24,65].

### 3.4. Physicochemical Properties, Sensory Attributes, and Proximate Composition of Optimized Cookies

Following numerical optimization and experimental validation of the response surface models, the physicochemical, sensory, and nutritional properties of cookies prepared with the optimized formulation (41% acorn flour, 1.5% xanthan gum) were evaluated and compared to the control cookies (50% rice flour, 50% maize flour, 1.5% xanthan gum). An independent samples *t*-test (*p* < 0.05) was used to determine statistically significant differences between the two formulations.

The significantly darker color of cookies with added acorn flour was confirmed by lower L* values (decreasing from 66.3 to 46.4, about 20 units) and higher *DI* (increasing from 51.7 to 60.2), with a simultaneous increase in a* and a decrease in b* values (Table 3). These results are consistent with the high content of phenolic compounds in acorn flour, including gallic and ellagic acids, catechins, and rutin, which contribute to Maillard and caramelization reactions during baking [77,78,79]. Hemmati Kaykha et al. (2025) [71] also reported that acorn flour is the main driver of color changes in gluten-free baked products, while hydrocolloids exert limited corrective effects. Despite the darker appearance, sensory evaluation showed no significant difference in visual acceptability (Table 4), indicating that the darkening was positively perceived as characteristic of wholegrain or artisanal products.

The addition of acorn flour significantly decreased the snapping force (~16%) and bending stiffness (~14%), reflecting a more brittle structure (Table 3). This is in agreement with the literature reporting that increased fiber and lipid content in gluten-free formulations disrupts the starch network, reducing mechanical resistance [22,80]. The small decrease in distance to break (1.20 → 1.17 mm) suggests that product handling is not adversely affected. The presence of unsaturated fatty acids in acorn flour may act as a “texture softener,” while insoluble fibers interrupt matrix continuity [77].

Width, thickness, and spread factor of the cookies did not differ significantly from the control (Table 3), indicating that hydrocolloids, particularly xanthan gum, stabilized the dough structure and limited expansion during baking [71,81]. These findings differ slightly from those of Pasqualone et al. (2019) [22], who reported increased cookie spread when wheat flour was partially replaced with acorn flour. In that study, higher levels of acorn flour and a corresponding reduction in wheat flour—and thus gluten content—resulted in weaker dough structure and greater spreading during baking. In contrast, the present formulation did not rely on gluten for structural stability but instead used hydrocolloids to compensate for the absence of gluten, which likely prevented excessive spreading despite the inclusion of acorn flour.

Water activity was statistically significantly higher in acorn cookies (0.47 compared to 0.43) (Table 3), likely due to the higher content of insoluble fiber, which binds water in the cookie matrix [70,77]. Moisture content (Table 5) remained within typical ranges for low-moisture cookies (4.26 → 4.53%), with no statistically significant differences between the control and optimized formulations. The observed water activity values were still below thresholds for microbial growth, indicating safe product stability.

Overall sensory acceptability remained high, with statistically significant improvement in taste scores (7.13 → 7.63) and no significant changes in appearance, color, texture, or odor (Table 5). These findings suggest that the phenolic compounds in acorn flour did not induce undesirable bitterness or astringency, consistent with reports by Hemmati Kaykha et al. (2025) [71] and Martins et al. (2022) [77]. The addition of acorn flour contributed to a richer flavor profile, likely enhancing roasted and caramel-like notes.

No significant differences were observed in the protein, fat, or carbohydrate content (Table 5), reflecting the relatively low protein content of acorn flour (~4.3%) [77]. Significant increases were observed in ash (1.70 → 1.96%) and total dietary fiber (1.52 → 3.51%), primarily due to insoluble fiber (0.62 → 2.48%). These results align with the literature reporting the high mineral and fiber content of acorn flour [1,82].

The optimized formulation (3.51 g/100 g fiber) meets the EU regulatory requirement for the nutrition claim “source of fibre” (≥3 g/100 g), although it does not reach the “high fibre” threshold (≥6 g/100 g). This is particularly relevant for gluten-free bakery products, which are often characterized by low fiber content.

The most pronounced changes were observed in the content of phenolic compounds and antioxidant capacity of the cookies (Table 6). Cookies with added acorn flour had about six times higher total phenolic content, increasing from 1.63 mg GAE/g in control cookies to 10.08 mg GAE/g in acorn cookies, and 8–10 times higher antioxidant potential, depending on the method used (DPPH, ABTS, and FRAP). These results align with the findings of [77], who reported that acorn flour contains high concentrations of gallic and ellagic acids, catechins, and rutin, and exhibits very high antioxidant potential compared to other gluten-free flours [1,83]. The retention of high antioxidant potential in cookies confirms the stability of acorn phenolic compounds during heat treatment, as previously reported in other bakery products [22,82,84].

**Table 6:** Polyphenol content and antioxidant properties of control (50% rice flour, 50% maize flour, 1.5% xanthan gum) and acorn cookies (41% acorn flour, 29.5% rice flour, 29.5% maize flour, 1.5% xanthan gum).

Parameter (Dry Matter)	Control Cookies ^1^	Acorn Cookies ^1^	*t*	*p*
Total phenolic content (mg GAE/g)	1.63 ± 0.33	10.08 ± 1.49	9.56	0.001 *
Non-tannin phenolic content (mg GAE/g)	1.54 ± 0.47	6.03 ± 0.48	11.66	<0.001 *
Tannin content (mg GAE/g)	0.09 ± 0.06	4.05 ± 0.99	6.90	0.002 *
DPPH radical scavenging activity (µmol TE/g)	12.70 ± 2.69	99.82 ± 3.81	32.34	<0.001 *
ABTS radical scavenging activity (µmol TE/g)	15.90 ± 2.30	140.38 ± 4.91	39.75	<0.001 *
FRAP antioxidant capacity (µmol TE/g)	14.26 ± 2.46	134.47 ± 7.75	25.60	<0.001 *

^1^ Values are mean ± SD. * *p* < 0.05; Student’s *t*-test.

The addition of 41% acorn flour enabled the development of functional gluten-free cookies with improved nutritional and antioxidant properties, without negatively affecting the sensory acceptability of the product. In accordance with the findings of Martins et al. (2022) [77] and Hemmati Kaykha et al. (2025) [71], the results confirm that properly formulated products with acorn flour can achieve a balance between technological quality, functionality, and consumer acceptance, confirming acorn as a valuable and sustainable raw material for the development of innovative gluten-free bakery products.

It is also important to note that this research used acorn flour without leaching tannins, as the study aimed to preserve other beneficial substances such as soluble proteins, fibers, and sugars, which can be lost during leaching. Therefore, future research may focus on using acorns from oaks with lower tannin content (e.g., *Q. rotundifolia*) to further increase the proportion of acorn flour in products.

## 4. Conclusions

This study demonstrated that *Quercus pubescens* acorn flour can be successfully incorporated into gluten-free cookies when formulation parameters are carefully optimized. RSM enabled a systematic evaluation of the combined effects of acorn flour and xanthan gum on technological, sensory, and functional attributes. Increasing the acorn flour content significantly affected cookie color, texture, and sensory perception. Higher substitution levels produced darker cookies, reduced snapping force and bending stiffness, and more pronounced nutty and earthy flavor notes, reflecting the high fiber and phenolic content of acorn flour. Xanthan gum played a crucial structural role, improving dough cohesion, dimensional stability, and mechanical resistance, particularly at intermediate concentrations, while excessive levels could negatively affect spread and texture. Multi-response optimization identified an optimal formulation containing approximately 41% acorn flour and 1.5% xanthan gum, representing the best compromise between structural integrity, water activity, color development, and trained-panel hedonic evaluation scores. Compared to control cookies, the optimized formulation showed improved nutritional and functional characteristics, including approximately double the dietary fiber content, increased mineral content (ash), and a sixfold increase in total phenolic content, accompanied by 8–10 times higher in vitro antioxidant capacity (DPPH, ABTS, FRAP assays). These enhancements indicate increased functional potential associated with acorn flour incorporation.

Overall, the results support the potential of *Q. pubescens* acorn flour as a sustainable, value-added ingredient for gluten-free bakery applications, contributing to the valorization of underutilized forest resources and the development of nutritionally enhanced gluten-free products.

## Figures and Tables

**Table 1:** Composition of raw materials used in gluten-free acorn cookies *(Q. pubescens*) based on the experimental design.

Raw Materials ^1^	Run 1	Run 2	Run 3	Run 4	Run 5	Run 6	Run 7	Run 8	Run 9	Run 10	Run 11	Run 12
Acorn flour (*A*)	100	50	-	100	50	-	100	50	-	50	50	50
Rice flour	-	25	50	-	25	50	-	25	50	25	25	25
Maize flour	-	25	50	-	25	50	-	25	50	25	25	25
Xanthan gum (*B*)	1	1	1	2	2	2	3	3	3	2	2	2
Whey protein	10											
Margarine	40											
Sugar	42											
NaCl	1.25											
NaHCO_3_	1											
NH_4_HCO_3_	1											
Water	22											

^1^ Raw materials are expressed as baker’s percentage. Whey protein, margarine, sugar, NaCl, NaHCO_3_, NH_4_HCO_3_, and water were kept constant across all experimental runs. Runs 5, 10, 11, and 12 are replicates of the central point (50% acorn flour, 2% xanthan gum) used to estimate experimental error and validate model adequacy in the RSM design. “-” indicates that the ingredient was not included in the formulation (0%, baker’s percentage).

**Table 2:** Model adequacy statistics for responses used in multi-response optimization of gluten-free acorn cookies.

Response	Model Type	*R* ^2^	Adjusted *R*^2^	Predicted *R*^2^	CV %
L*	Quadratic	0.9983	0.9968	0.9949	1.47
a*	Linear	0.9251	0.9084	0.8784	1.48
b*	Quadratic	0.9955	0.9917	0.9870	3.09
Darkness index	Quadratic	0.9959	0.9925	0.9885	0.881
Snapping force (N)	Linear	0.9791	0.9745	0.9584	3.56
Distance to break (mm)	Linear	0.8576	0.8260	0.7890	1.99
Bending stiffness (N/mm)	Linear	0.9833	0.9796	0.9659	2.74
Width (cm)	Quadratic	0.9728	0.9502	0.7383	0.395
Thickness (cm)	Quadratic	0.8771	0.7747	0.7674	1.72
Spread factor	Quadratic	0.9317	0.8748	0.8393	1.74
Water content (%)	Quadratic	0.9529	0.9137	0.9098	1.97
*a_w_*	Quadratic	0.9792	0.9619	0.9425	1.15
Appearance	Quadratic	0.9793	0.9621	0.8984	1.09
Color	Quadratic	0.8967	0.8106	0.4617	1.46
Texture	Quadratic	0.9475	0.9037	0.8589	1.46
Odor	Quadratic	0.9653	0.9364	0.8745	1.87
Taste	Quadratic	0.9821	0.9671	0.9353	1.56
Overall acceptability	Quadratic	0.9688	0.9427	0.8201	1.13

L*—lightness; a*—red–green coordinate; b*—yellow–blue coordinate; *a_w_*—water activity; *R*^2^—coefficient of determination; Adjusted *R*^2^—adjusted coefficient of determination; Predicted *R*^2^—predicted coefficient of determination; CV %—coefficient of variation.

**Table 3:** Physical properties of control (50% rice flour, 50% maize flour, 1.5% xanthan gum) and acorn cookies (41% acorn flour, 29.5% rice flour, 29.5% maize flour, 1.5% xanthan gum).

Parameter	Control Cookies ^1^	Acorn Cookies ^1^	*t*	*p*
L*	66.30 ± 0.90	46.40 ± 0.62	−31.46	<0.001 *
a*	14.63 ± 0.15	15.30 ± 0.20	4.59	0.010 *
b*	36.33 ± 0.06	22.67 ± 0.78	−30.39	<0.001 *
Darkness index	51.67 ± 0.67	60.18 ± 0.57	16.78	<0.001 *
Snapping force (N)	51.20 ± 3.17	42.90 ± 1.77	−3.96	0.017 *
Distance to break (mm)	1.20 ± 0.01	1.17 ± 0.02	−3.16	0.034 *
Bending stiffness (N/mm)	42.66 ± 2.40	36.76 ± 1.07	−3.88	0.018 *
Width (cm)	4.84 ± 0.07	4.77 ± 0.06	−1.33	0.253
Thickness (cm)	1.05 ± 0.01	1.05 ± 0.01	−0.50	0.643
Spread factor	46.10 ± 0.22	45.43 ± 0.27	−2.22	0.091
*a_w_*	0.43 ± 0.00	0.47 ± 0.01	4.72	0.009 *

^1^ Values are mean ± SD. L*—lightness; a*—red–green coordinate; b*—yellow–blue coordinate; *a_w_*—water activity. * *p* < 0.05; Student’s *t*-test.

**Table 4:** Sensory properties of control (50% rice flour, 50% maize flour, 1.5% xanthan gum) and acorn cookies (41% acorn flour, 29.5% rice flour, 29.5% maize flour, 1.5% xanthan gum).

Parameter	Control Cookies ^1^	Acorn Cookies ^1^	*t*	*p*
Appearance	8.03 ± 0.12	7.90 ± 0.17	−1.11	0.329
Color	8.03 ± 0.25	8.00 ± 0.10	−0.21	0.842
Texture	7.50 ± 0.30	7.70 ± 0.17	1.00	0.374
Odor	7.77 ± 0.21	8.00 ± 0.10	1.75	0.155
Taste	7.13 ± 0.15	7.63 ± 0.15	4.01	0.016 *
Overall acceptability	7.50 ± 0.30	7.87 ± 0.12	1.98	0.119

^1^ Values are mean ± SD. * *p* < 0.05; Student’s *t*-test.

**Table 5:** Proximate composition of control (50% rice flour, 50% maize flour, 1.5% xanthan gum) and acorn cookies (41% acorn flour, 29.5% rice flour, 29.5% maize flour, 1.5% xanthan gum).

Parameter (As Is)	Control Cookies ^1^	Acorn Cookies ^1^	*t*	*p*
Moisture (%)	4.26 ± 0.16	4.53 ± 0.23	1.66	0.173
Protein (%)	8.39 ± 0.34	8.01 ± 0.29	−1.46	0.219
Fat (%)	18.53 ± 1.51	19.26 ± 1.11	0.67	0.537
Ash (%)	1.70 ± 0.09	1.96 ± 0.13	2.82	0.048 *
Carbohydrates (%)	67.12 ± 1.12	66.24 ± 0.97	−1.03	0.362
Total sugars (%)	23.51 ± 1.51	25.30 ± 1.13	1.65	0.175
Soluble fiber (%)	0.90 ± 0.11	1.03 ± 0.17	1.10	0.331
Insoluble fiber (%)	0.62 ± 0.16	2.48 ± 0.26	10.65	<0.001 *
Total fiber (%)	1.52 ± 0.08	3.51 ± 0.13	22.31	<0.001 *

^1^ Values are mean ± SD. * *p* < 0.05; Student’s *t*-test.

## Data Availability

The original contributions presented in this study are included in the article/Supplementary Materials. Further inquiries can be directed to the corresponding author.

## Supplementary Materials

The following supporting information can be downloaded at https://www.mdpi.com/article/10.3390/foods15050966/s1. Table S1. Analysis of variance (ANOVA) for the quadratic model describing the effect of acorn flour and xanthan gum on *L** (lightness) of gluten-free acorn cookies; Table S2. Analysis of variance (ANOVA) for the linear model describing the effect of acorn flour and xanthan gum on *a** (red–green coordinate) of gluten-free acorn cookies; Table S3. Analysis of variance (ANOVA) for the quadratic model describing the effect of acorn flour and xanthan gum on *b** (yellow–blue coordinate) of gluten-free acorn cookies; Table S4. Analysis of variance (ANOVA) for the quadratic model describing the effect of acorn flour and xanthan gum on the darkness index (*DI*) of gluten-free acorn cookies; Table S5. Analysis of variance (ANOVA) for the linear model describing the effect of acorn flour and xanthan gum on snapping force of gluten-free acorn cookies; Table S6. Analysis of variance (ANOVA) for the linear model describing the effect of acorn flour and xanthan gum on distance to break of gluten-free acorn cookies; Table S7. Analysis of variance (ANOVA) for the linear model describing the effect of acorn flour and xanthan gum on bending stiffness of gluten-free acorn cookies; Table S8. Analysis of variance (ANOVA) for the quadratic model describing the effect of acorn flour and xanthan gum on cookie width; Table S9. Analysis of variance (ANOVA) for the quadratic model describing the effect of acorn flour and xanthan gum on cookie thickness; Table S10. Analysis of variance (ANOVA) for the quadratic model describing the effect of acorn flour and xanthan gum on the spread factor of gluten-free acorn cookies; Table S11. Analysis of variance (ANOVA) for the quadratic model describing the effect of acorn flour and xanthan gum on water content of gluten-free acorn cookies; Table S12. Analysis of variance (ANOVA) for the quadratic model describing the effect of acorn flour and xanthan gum on water activity (*a_w_*) of gluten-free acorn cookies; Table S13. Analysis of variance (ANOVA) for the quadratic model describing the effect of acorn flour and xanthan gum on sensory appearance scores of gluten-free acorn cookies; Table S14. Analysis of variance (ANOVA) for the quadratic model describing the effect of acorn flour and xanthan gum on sensory color scores of gluten-free acorn cookies; Table S15. Analysis of variance (ANOVA) for the quadratic model describing the effect of acorn flour and xanthan gum on sensory texture scores of gluten-free acorn cookies; Table S16. Analysis of variance (ANOVA) for the quadratic model describing the effect of acorn flour and xanthan gum on sensory odor scores of gluten-free acorn cookies; Table S17. Analysis of variance (ANOVA) for the quadratic model describing the effect of acorn flour and xanthan gum on sensory taste scores of gluten-free acorn cookies; Table S18. Analysis of variance (ANOVA) for the quadratic model describing the effect of acorn flour and xanthan gum on overall acceptability of gluten-free acorn cookies.

## Abbreviations

The following abbreviations are used in this manuscript:

*a**	Red–green coordinate (CIELAB)
ABTS	2,2′-azino-bis(3-ethylbenzothiazoline-6-sulfonic acid)(radical cation assay)
ANOVA	Analysis of variance
AOAC	Association of Official Agricultural Chemists
*a_w_*	Water activity
*b**	Yellow–blue coordinate (CIELAB)
CIE	Commission Internationale de l’Éclairage (International Commission on Illumination)
CV %	Coefficient of variation
*DI*	Darkness index
DPPH	2,2-diphenyl-1-picrylhydrazyl (radical scavenging assay)
FFD	Full factorial design
FRAP	Ferric reducing antioxidant power
GAE	Gallic acid equivalent
GF	Gluten-free
*H*	Thickness
HPMC	Hydroxypropyl methylcellulose
ISO	International Organization for Standardization
*L**	Lightness (CIELAB)
NTPC	Non-tannin phenolic compounds
PI	Prediction interval
PVPP	Polyvinylpolypyrrolidone
*R*^2^	Coefficient of determination
RSM	Response surface methodology
*SD*	Standard deviation
*SF*	Spread factor
TAE	Tannic acid equivalent
TE	Trolox equivalent
TP	Polyphenol
TPC	Total polyphenol content
TPE	Total polyphenol extract
TPC-PVPP	Total polyphenols after PVPP treatment (tannin-depleted fraction)
TPF	Total polyphenol fraction
TPTZ	2,4,6-tris(2-pyridyl)-s-triazine
*W*	Width
