# Supplementary material for: Application of Quercus pubescens Acorn Flour and Xanthan Gum in Gluten-Free Cookies: RSM Optimization and Quality Evaluation
Source: Foods. 2026 Mar 9;15(5):966. doi: 10.3390/foods15050966 (PMC12984973; doi:10.3390/foods15050966)
Supplement: Supplementary file 1 [file foods-15-00966-s001.zip › foods-4161427-supplementary.pdf]

Table S1. Analysis of variance (ANOVA) for the quadratic model describing the effect of acorn flour and xanthan gum on ***L**(lightness)** of gluten-free acorn cookies.

Source of variation	SS	df	MS	*F*	*p*	
**Model**	1568.07	5	313.61	693.82	**< 0.0001**	significant
A-Acorn flour (%)	1320.17	1	1320.17	2920.63	< 0.0001	
B-Xanthan gum (%)	11.76	1	11.76	26.02	0.0022	
AB	0.7225	1	0.7225	1.60	0.2530	
A^2	202.42	1	202.42	447.82	< 0.0001	
B^2	0.4538	1	0.4538	1.00	0.3551	
**Residual**	2.71	6	0.4520			
Lack of Fit	0.4621	3	0.1540	0.2054	0.8869	not significant
Pure Error	2.25	3	0.7500			
**Cor Total**	1570.78	11				

*L**—lightness in the CIE*L***a***b** color system; A—acorn flour (%); B—xanthan gum (%); AB—interaction effect; A^2, B^2—quadratic terms; SS—Sum of Squares; df—degrees of freedom; MS—Mean Squares. *F*—value is the model significance (regression/residual). Significance was evaluated at $p < 0.05$.

Table S2. Analysis of variance (ANOVA) for the linear model describing the effect of acorn flour and xanthan gum on ***a** (red–green coordinate)** of gluten-free acorn cookies.

Source of variation	SS	df	MS	*F*	*p*	
**Model**	5.74	2	2.87	55.56	< 0.0001	significant
A-Acorn flour (%)	4.86	1	4.86	94.06	< 0.0001	
B-Xanthan gum (%)	0.8817	1	0.8817	17.06	0.0026	
**Residual**	0.4650	9	0.0517			
Lack of Fit	0.2150	6	0.0358	0.4300	0.8256	not significant
Pure Error	0.2500	3	0.0833			
**Cor Total**	6.21	11				

*a**— red–green coordinate in the CIE*L***a***b** color system; A—acorn flour (%); B—xanthan gum (%); SS—Sum of Squares; df—degrees of freedom; MS—Mean Squares. *F*—value is the model significance (regression/residual). Significance was evaluated at $p < 0.05$.

Table S3. Analysis of variance (ANOVA) for the quadratic model describing the effect of acorn flour and xanthan gum on ***b** (yellow–blue coordinate)** of gluten-free acorn cookies.

Source of variation	SS	df	MS	*F*	*p*	
**Model**	692.90	5	138.58	263.79	< 0.0001	significant
A-Acorn flour (%)	598.00	1	598.00	1138.30	< 0.0001	
B-Xanthan gum (%)	2.28	1	2.28	4.34	0.0823	
AB	0.2025	1	0.2025	0.3855	0.5575	
A^2	81.77	1	81.77	155.65	< 0.0001	
B^2	0.0038	1	0.0038	0.0071	0.9354	
**Residual**	3.15	6	0.5253			
Lack of Fit	0.4621	3	0.1540	0.1718	0.9090	not significant
Pure Error	2.69	3	0.8967			
**Cor Total**	696.05	11				

*L**—lightness in the CIE*L***a***b** color system; A—acorn flour (%); B—xanthan gum (%); AB—interaction effect; A^2, B^2—quadratic terms; SS—Sum of Squares; df—degrees of freedom; MS—Mean Squares. *F*—value is the model significance (regression/residual). Significance was evaluated at $p < 0.05$.

Table S4. Analysis of variance (ANOVA) for the quadratic model describing the effect of acorn flour and xanthan gum on the **darkness index (*DI*)** of gluten-free acorn cookies.

Source of variation	SS	df	MS	*F*	*p*	
**Model**	437.59	5	87.52	291.48	< 0.0001	significant
A-Acorn flour (%)	392.87	1	392.87	1308.45	< 0.0001	
B-Xanthan gum (%)	5.39	1	5.39	17.94	0.0055	
AB	0.0106	1	0.0106	0.0353	0.8571	
A^2	32.73	1	32.73	109.01	< 0.0001	
B^2	0.2645	1	0.2645	0.8810	0.3842	
**Residual**	1.80	6	0.3003			
Lack of Fit	0.2937	3	0.0979	0.1948	0.8939	not significant
Pure Error	1.51	3	0.5026			
**Cor Total**	439.40	11				

DI— darkness index; A—acorn flour (%); B—xanthan gum (%); AB—interaction effect; A^2, B^2—quadratic terms; SS—Sum of Squares; df—degrees of freedom; MS—Mean Squares. *F*—value is the model significance (regression/residual). Significance was evaluated at $p < 0.05$.

Table S5. Analysis of variance (ANOVA) for the linear model describing the effect of acorn flour and xanthan gum on **snapping force** of gluten-free acorn cookies.

Source of variation	SS	df	MS	*F*	*p*	
**Model**	1115.56	2	557.78	210.88	< 0.0001	significant
A-Acorn flour (%)	873.63	1	873.63	330.29	< 0.0001	
B-Xanthan gum (%)	241.93	1	241.93	91.47	< 0.0001	
**Residual**	23.81	9	2.65			
Lack of Fit	12.71	6	2.12	0.5723	0.7434	not significant
Pure Error	11.10	3	3.70			
**Cor Total**	1139.37	11				

A—acorn flour (%); B—xanthan gum (%); AB—interaction effect; SS—Sum of Squares; df—degrees of freedom; MS—Mean Squares. *F*—value is the model significance (regression/residual). Significance was evaluated at $p < 0.05$.

Table S6. Analysis of variance (ANOVA) for the linear model describing the effect of acorn flour and xanthan gum on **distance to break** of gluten-free acorn cookies.

Source of variation	SS	df	MS	*F*	*p*	
**Model**	0.0304	2	0.0152	27.10	0.0002	significant
A-Acorn flour (%)	0.0037	1	0.0037	6.68	0.0294	
B-Xanthan gum (%)	0.0267	1	0.0267	47.52	< 0.0001	
**Residual**	0.0051	9	0.0006			
Lack of Fit	0.0025	6	0.0004	0.4712	0.8013	not significant
Pure Error	0.0026	3	0.0009			
**Cor Total**	0.0355	11				

A—acorn flour (%); B—xanthan gum (%); SS—Sum of Squares; df—degrees of freedom; MS—Mean Squares. *F*—value is the model significance (regression/residual). Significance was evaluated at $p < 0.05$.

Table S7. Analysis of variance (ANOVA) for the linear model describing the effect of acorn flour and xanthan gum on **bending stiffness** of gluten-free acorn cookies.

Source of variation	SS	df	MS	*F*	*p*	
**Model**	577.47	2	288.73	265.48	< 0.0001	significant
A-Acorn flour (%)	516.15	1	516.15	474.58	< 0.0001	
B-Xanthan gum (%)	61.32	1	61.32	56.38	< 0.0001	
**Residual**	9.79	9	1.09			
Lack of Fit	8.20	6	1.37	2.58	0.2340	not significant
Pure Error	1.59	3	0.5304			
**Cor Total**	587.26	11				

A—acorn flour (%); B—xanthan gum (%); SS—Sum of Squares; df—degrees of freedom; MS—Mean Squares. *F*—value is the model significance (regression/residual). Significance was evaluated at $p < 0.05$.

Table S8. Analysis of variance (ANOVA) for the quadratic model describing the effect of acorn flour and xanthan gum on cookie **width**.

Source of variation	SS	df	MS	*F*	*p*	
**Model**	0.0746	5	0.0149	42.96	0.0001	significant
A-Acorn flour (%)	0.0417	1	0.0417	120.00	< 0.0001	
B-Xanthan gum (%)	0.0267	1	0.0267	76.80	0.0001	
AB	0.0025	1	0.0025	7.20	0.0364	
A^2	0.0004	1	0.0004	1.20	0.3153	
B^2	0.0038	1	0.0038	10.80	0.0167	
Residual	0.0021	6	0.0003			
Lack of Fit	0.0021	3	0.0007			
Pure Error	0.0000	3	0.0000			
**Cor Total**	0.0767	11				

A—acorn flour (%); B—xanthan gum (%); AB—interaction effect; A^2, B^2—quadratic terms; SS—Sum of Squares; df—degrees of freedom; MS—Mean Squares. *F*—value is the model significance (regression/residual). Significance was evaluated at $p < 0.05$.

Table S9. Analysis of variance (ANOVA) for the quadratic model describing the effect of acorn flour and xanthan gum on cookie **thickness**.

Source of variation	SS	df	MS	*F*	*p*	
**Model**	0.0140	5	0.0028	8.56	0.0105	significant
A-Acorn flour (%)	0.0000	1	0.0000	0.0508	0.8291	
B-Xanthan gum (%)	0.0104	1	0.0104	31.78	0.0013	
AB	0.0009	1	0.0009	2.75	0.1486	
A^2	0.0000	1	0.0000	0.0000	1.0000	
B^2	0.0024	1	0.0024	7.32	0.0353	
Residual	0.0020	6	0.0003			
Lack of Fit	0.0001	3	0.0000	0.0351	0.9895	not significant
Pure Error	0.0019	3	0.0006			
**Cor Total**	0.0160	11				

A—acorn flour (%); B—xanthan gum (%); AB—interaction effect; A^2, B^2—quadratic terms; SS—Sum of Squares; df—degrees of freedom; MS—Mean Squares. *F*—value is the model significance (regression/residual). Significance was evaluated at $p < 0.05$.

Table S10. Analysis of variance (ANOVA) for the quadratic model describing the effect of acorn flour and xanthan gum on the **spread factor** of gluten-free acorn cookies.

**Source of variation**	**SS**	**df**	**MS**	***F***	***p***	
**Model**	50.39	5	10.08	16.38	0.0019	significant
A-Acorn flour (%)	4.14	1	4.14	6.73	0.0409	
B-Xanthan gum (%)	37.00	1	37.00	60.13	0.0002	
AB	0.6258	1	0.6258	1.02	0.3521	
A^2	0.0455	1	0.0455	0.0739	0.7948	
B^2	8.02	1	8.02	13.04	0.0112	
Residual	3.69	6	0.6153			
Lack of Fit	0.3347	3	0.1116	0.0997	0.9549	not significant
Pure Error	3.36	3	1.12			
**Cor Total**	54.08	11				

A—acorn flour (%); B—xanthan gum (%); AB—interaction effect; A^2, B^2—quadratic terms; SS—Sum of Squares; df—degrees of freedom; MS—Mean Squares. *F*—value is the model significance (regression/residual). Significance was evaluated at $p < 0.05$.

Table S11. Analysis of variance (ANOVA) for the quadratic model describing the effect of acorn flour and xanthan gum on **water content** of gluten-free acorn cookies.

**Source of variation**	**SS**	**df**	**MS**	***F***	***p***	
**Model**	1.05	5	0.2109	24.30	0.0006	significant
A-Acorn flour (%)	0.6667	1	0.6667	76.80	0.0001	
B-Xanthan gum (%)	0.2817	1	0.2817	32.45	0.0013	
AB	0.0025	1	0.0025	0.2880	0.6108	
A^2	0.0204	1	0.0204	2.35	0.1760	
B^2	0.0504	1	0.0504	5.81	0.0526	
**Residual**	0.0521	6	0.0087			
Lack of Fit	0.0021	3	0.0007	0.0417	0.9866	not significant
Pure Error	0.0500	3	0.0167			
**Cor Total**	1.11	11				

A—acorn flour (%); B—xanthan gum (%); AB—interaction effect; A^2, B^2—quadratic terms; SS—Sum of Squares; df—degrees of freedom; MS—Mean Squares. *F*—value is the model significance (regression/residual). Significance was evaluated at $p < 0.05$.

Table S12. Analysis of variance (ANOVA) for the quadratic model describing the effect of acorn flour and xanthan gum on **water activity (a_w)** of gluten-free acorn cookies.

**Source of variation**	**SS**	**df**	**MS**	***F***	***p***	
**Model**	0.0085	5	0.0017	56.59	< 0.0001	significant
A-Acorn flour (%)	0.0046	1	0.0046	152.74	< 0.0001	
B-Xanthan gum (%)	0.0031	1	0.0031	104.03	< 0.0001	
AB	0.0000	1	0.0000	0.6734	0.4432	
A^2	0.0002	1	0.0002	6.41	0.0446	
B^2	0.0003	1	0.0003	10.73	0.0169	
**Residual**	0.0002	6	0.0000			
Lack of Fit	0.0000	3	9.889E-06	0.1968	0.8926	not significant
Pure Error	0.0002	3	0.0001			
**Cor Total**	0.0087	11				

A—acorn flour (%); B—xanthan gum (%); AB—interaction effect; A^2, B^2—quadratic terms; SS—Sum of Squares; df—degrees of freedom; MS—Mean Squares. *F*—value is the model significance (regression/residual). Significance was evaluated at $p < 0.05$.

Table S13. Analysis of variance (ANOVA) for the quadratic model describing the effect of acorn flour and xanthan gum on sensory **appearance** scores of gluten-free acorn cookies.

Source of variation	SS	df	MS	*F*	*p*	
**Model**	2.11	5	0.4224	56.85	< 0.0001	significant
A-Acorn flour (%)	1.60	1	1.60	215.55	< 0.0001	
B-Xanthan gum (%)	0.1667	1	0.1667	22.43	0.0032	
AB	0.0000	1	0.0000	0.0000	1.0000	
A^2	0.2604	1	0.2604	35.05	0.0010	
B^2	0.0104	1	0.0104	1.40	0.2812	
**Residual**	0.0446	6	0.0074			
Lack of Fit	0.0171	3	0.0057	0.6212	0.6474	not significant
Pure Error	0.0275	3	0.0092			
**Cor Total**	2.16	11				

A—acorn flour (%); B—xanthan gum (%); AB—interaction effect; A^2, B^2—quadratic terms; SS—Sum of Squares; df—degrees of freedom; MS—Mean Squares. *F*—value is the model significance (regression/residual). Significance was evaluated at $p < 0.05$.

Table S14. Analysis of variance (ANOVA) for the quadratic model describing the effect of acorn flour and xanthan gum on sensory **color** scores of gluten-free acorn cookies.

Source of variation	SS	df	MS	*F*	*p*	
**Model**	0.7054	5	0.1411	10.42	0.0064	significant
A-Acorn flour (%)	0.4817	1	0.4817	35.57	0.0010	
B-Xanthan gum (%)	0.0600	1	0.0600	4.43	0.0799	
AB	0.0000	1	0.0000	0.0000	1.0000	
A^2	0.1504	1	0.1504	11.11	0.0158	
B^2	0.0004	1	0.0004	0.0308	0.8665	
**Residual**	0.0812	6	0.0135			
Lack of Fit	0.0338	3	0.0113	0.7105	0.6072	not significant
Pure Error	0.0475	3	0.0158			
**Cor Total**	0.7867	11				

A—acorn flour (%); B—xanthan gum (%); AB—interaction effect; A^2, B^2—quadratic terms; SS—Sum of Squares; df—degrees of freedom; MS—Mean Squares. *F*—value is the model significance (regression/residual). Significance was evaluated at $p < 0.05$.

Table S15. Analysis of variance (ANOVA) for the quadratic model describing the effect of acorn flour and xanthan gum on sensory **texture** scores of gluten-free acorn cookies.

Source of variation	SS	df	MS	*F*	*p*	
**Model**	1.24	5	0.2481	21.65	0.0009	significant
A-Acorn flour (%)	0.5400	1	0.5400	47.13	0.0005	
B-Xanthan gum (%)	0.0267	1	0.0267	2.33	0.1780	
AB	0.0025	1	0.0025	0.2182	0.6569	
A^2	0.3037	1	0.3037	26.51	0.0021	
B^2	0.1504	1	0.1504	13.13	0.0111	
**Residual**	0.0687	6	0.0115			
Lack of Fit	0.0087	3	0.0029	0.1458	0.9259	not significant
Pure Error	0.0600	3	0.0200			
**Cor Total**	1.31	11				

A—acorn flour (%); B—xanthan gum (%); AB—interaction effect; A^2, B^2—quadratic terms; SS—Sum of Squares; df—degrees of freedom; MS—Mean Squares. *F*—value is the model significance (regression/residual). Significance was evaluated at $p < 0.05$.

Table S16. Analysis of variance (ANOVA) for the quadratic model describing the effect of acorn flour and xanthan gum on sensory **odor** scores of gluten-free acorn cookies.

Source of variation	SS	df	MS	*F*	*p*	
**Model**	3.46	5	0.6929	33.37	0.0003	significant
A-Acorn flour (%)	0.8817	1	0.8817	42.46	0.0006	
B-Xanthan gum (%)	0.0417	1	0.0417	2.01	0.2064	
AB	0.0000	1	0.0000	0.0000	1.0000	
A^2	2.10	1	2.10	101.16	< 0.0001	
B^2	0.0204	1	0.0204	0.9833	0.3597	
**Residual**	0.1246	6	0.0208			
Lack of Fit	0.0371	3	0.0124	0.4238	0.7504	not significant
Pure Error	0.0875	3	0.0292			
**Cor Total**	3.59	11				

A—acorn flour (%); B—xanthan gum (%); AB—interaction effect; A^2, B^2—quadratic terms; SS—Sum of Squares; df—degrees of freedom; MS—Mean Squares. *F*—value is the model significance (regression/residual). Significance was evaluated at $p < 0.05$.

Table S17. Analysis of variance (ANOVA) for the quadratic model describing the effect of acorn flour and xanthan gum on sensory **taste** scores of gluten-free acorn cookies.

Source of variation	SS	df	MS	*F*	*p*	
**Model**	4.15	5	0.8307	65.72	< 0.0001	significant
A-Acorn flour (%)	0.4817	1	0.4817	38.11	0.0008	
B-Xanthan gum (%)	0.0067	1	0.0067	0.5275	0.4950	
AB	0.0625	1	0.0625	4.95	0.0678	
A^2	2.80	1	2.80	221.67	< 0.0001	
B^2	0.0817	1	0.0817	6.46	0.0440	
**Residual**	0.0758	6	0.0126			
Lack of Fit	0.0283	3	0.0094	0.5965	0.6592	not significant
Pure Error	0.0475	3	0.0158			
**Cor Total**	4.23	11				

A—acorn flour (%); B—xanthan gum (%); AB—interaction effect; A^2, B^2—quadratic terms; SS—Sum of Squares; df—degrees of freedom; MS—Mean Squares. *F*—value is the model significance (regression/residual). Significance was evaluated at $p < 0.05$.

Table S18. Analysis of variance (ANOVA) for the quadratic model describing the effect of acorn flour and xanthan gum on **overall acceptability** of gluten-free acorn cookies.

Source of variation	SS	df	MS	*F*	*p*	
**Model**	1.34	5	0.2687	37.20	0.0002	significant
A-Acorn flour (%)	0.3267	1	0.3267	45.23	0.0005	
B-Xanthan gum (%)	0.0267	1	0.0267	3.69	0.1030	
AB	0.0100	1	0.0100	1.38	0.2839	
A^2	0.3267	1	0.3267	45.23	0.0005	
B^2	0.3267	1	0.3267	45.23	0.0005	
**Residual**	0.0433	6	0.0072			
Lack of Fit	0.0233	3	0.0078	1.17	0.4511	not significant
Pure Error	0.0200	3	0.0067			
**Cor Total**	1.39	11				

A—acorn flour (%); B—xanthan gum (%); AB—interaction effect; A^2, B^2—quadratic terms; SS—Sum of Squares; df—degrees of freedom; MS—Mean Squares. *F*—value is the model significance (regression/residual). Significance was evaluated at $p < 0.05$.
